# Locating induced earthquakes with a network of seismic stations in Oklahoma via a deep learning method

**DOI:** 10.1038/s41598-020-58908-5

**Published:** 2020-02-06

**Authors:** Xiong Zhang, Jie Zhang, Congcong Yuan, Sen Liu, Zhibo Chen, Weiping Li

**Affiliations:** 1 School of Earth and Space Sciences, University of Science and Technology of China, Hefei, Anhui 230026 P. R. China; 2 School of Information Science and Technology, University of Science and Technology of China, Hefei, Anhui 230026 P. R. China

**Keywords:** Natural hazards, Seismology, Information technology

## Abstract

The accurate and automated determination of small earthquake (M_L_ < 3.0) locations is still a challenging endeavor due to low signal-to-noise ratio in data. However, such information is critical for monitoring seismic activity and assessing potential hazards. In particular, earthquakes caused by industrial injection have become a public concern, and regulators need a solid capability for estimating small earthquakes that may trigger the action requirements for operators to follow in real time. In this study, we develop a fully convolutional network and locate earthquakes induced during oil and gas operations in Oklahoma with data from 30 network stations. The network is trained by 1,013 cataloged events (M_L_ ≥ 3.0) as base data along with augmented data accounting for smaller events (3.0 > M_L_ ≥ 0.5), and the output is a 3D volume of the event location probability in the Earth. The prediction results suggest that the mean epicenter errors of the testing events (M_L_ ≥ 1.5) vary from 3.7 to 6.4 km, meeting the need of the traffic light system in Oklahoma, but smaller events (M_L_ = 1.0, 0.5) show errors larger than 11 km. Synthetic tests suggest that the accuracy of ground truth from catalog affects the prediction results. Correct ground truth leads to a mean epicenter error of 2.0 km in predictions, but adding a mean location error of 6.3 km to ground truth causes a mean epicenter error of 4.9 km. The automated system is able to distinguish certain interfered events or events out of the monitoring zone based on the output probability estimate. It requires approximately one hundredth of a second to locate an event without the need for any velocity model or human interference.

## Introduction

Locating earthquakes constitutes a fundamental problem in seismology^1,2^. In particular, the reporting of earthquake locations (or hypocenters) in real time helps provide an assessment of potential hazards in local areas. For moderate to large earthquakes, such real-time reporting could lead to the issuance of early warnings to the public prior to the arrival of destructive and deadly seismic waves; for small earthquakes, it helps characterize subsurface activities and delineate fault movements. An earthquake occurs when two blocks within the earth suddenly slip past one another. In addition to tectonism, seismicity can be induced by the addition or removal of either surface water or groundwater and by the injection or removal of fluids due to industrial activity^3,4^. In Netherlands, there have been over 1000 induced earthquakes in the Groningen gas field since 1986. An M_L_ 3.6 event on 16 August 2012 caused building damage and led to serious public concern^5^. In China, along with increasing seismicity, several moderate earthquakes (M_L_ 4.4-5.7) occurred in the shale gas production field in Sichuan Basin. Three recent earthquakes of M_L_ 4.4-4.9 on 24 and 25 February 2019 in the field caused two deaths, 12 injuries, and damage to 1,091 houses. In Oklahoma, US, approximately 900 widely felt M ≥ 3.0 earthquakes occurred in north-central region in 2015, while only one M ≥ 3.0 earthquake occurred in Oklahoma on average each year before 2009^4,6^. It is now widely recognized that this almost 900-fold increase in earthquake occurrence is related to the widespread disposal of saltwater being coproduced with oil in seismically active areas^3,4^. In addition, the largest earthquake reported to date to be induced by fluid injection is the 2016 M 5.8 Pawnee, Oklahoma earthquake^7^. Oklahoma regulators launched a traffic-light system in 2016 requiring operators to take action if an event of M_L_ ≥ 2.5 occurs, which was further lowered to M_L_ ≥ 2.0 in 2018^8^. Oklahoma regulators also realized that they need to build an automated system capable of reporting earthquakes 24 hours a day to meet the protocol, but the current practice is to verify earthquakes manually by an analyst during normal business hours^8^. Similar traffic-light protocols were also developed in several other states in the US or other countries where induced earthquakes are concerned^3,7^. Therefore, there is a strong demand for technology that can timely and accurately report small earthquakes automatically, providing regulators with a firm scientific foundation for establishing requirements under which industry operates, and giving public assurance that the regulations are adequate and are being followed^3^.

Earthquakes are conventionally located through a process composed of detecting events, picking the arrival times of P-waves, and estimating the hypocentral parameters from the arrival times using a velocity model. Picking the first arrivals may also serve as event detection. Moreover, picks of P-wave arrival times from two or more seismic stations are needed to locate an event. Utilizing arrival times to locate earthquakes as opposed to waveforms simplifies the problem considerably; the corresponding methods, which include travel time inversion^9^, grid search^10^, and double-difference techniques^11^, are implemented in many different forms. However, conventional arrival time methods suffer from uncertainties in the time picks, especially for events with lower magnitude (M < 3). Thus, the dependence on human verification often delays the availability of the results.

Three-component waveform data should contain more earthquake information than only the arrival times of P-waves. Significant advance has been made in utilizing waveform data in seismological studies. These include many recent efforts of applying artificial intelligence to detect events or signals^12–21^ and pick seismic phases^22–24^. However, utilizing waveform data to locate earthquakes in a large area is challenging because numerous parameters influence seismic data in addition to the hypocentral parameters. Current efforts for locating earthquakes with machine learning methods are either limited to data from a single station^21,25^ or a small area with a few labels^15,26,27^. Among limited efforts to develop an automated detection system for a general earthquake location problem, an earthquake search engine method that applies fast search algorithms in computer science was introduced to find the best match for an earthquake waveform from a preset synthetic database, thereby returning the source information from the matched synthetic within a second^28^. This method is robust for dealing with long-period data at a large recording scale, but it is difficult to implement for regional or local earthquake monitoring, since the waveform data for which are highly sensitive to structural heterogeneities. Recently, another attempt was performed to apply artificial intelligence, specifically, the convolutional neural network (CNN) method, to detect seismic events from streaming waveform data^21^. This method can detect more than 17 times more earthquakes than a catalog by using single-station data in real-time applications, and it also outputs the probabilistic locations of detected events. However, CNN methods that implement the multilabel classification of training data from single-station waveforms could only approximately map induced seismicity in Oklahoma into six large areas. Unfortunately, while these probabilistic surface locations are helpful, they are not comparable to the hypocenter accuracy required for earthquake catalogs^29^.

In this study, we focus on real-time earthquake location problems for small earthquakes by accessing seismic waveform data from a regional network of 30 stations in Oklahoma. Motivated by the recent success of applying CNNs to solve inverse problems in medical imaging^30^, we design a novel architecture, namely, the fully convolutional network (FCN), which can predict a 3D image of the earthquake location probability in the Earth from a volume of input data recorded at multiple network stations. The FCN is initially developed for image segmentation^31,32^, medical image reconstruction^33^, and synthesizing high-quality images from text descriptions^34^, where the output is also a pixel image representing the recognized object position in the input. This approach is different from the typical application of CNNs to classification tasks, where the output for the input data is a single class label. Instead, the output of our network includes a large number of pixels representing a 3D image, in which the peak value corresponds to the most likely source location in the Earth. A deep learning approach for locating small earthquakes is appealing because it does not need picking.

To monitor the induced seismicity in Oklahoma, 1,013 cataloged earthquakes (M_L_ ≥ 3.0) are used as base training samples, and further constructed data (5 times of the base data) from these training samples are augmented in the training set to account for smaller events (3.0 > M_L_ ≥ 0.5). We tested events in four separate magnitude groups: (1) ML ≥ 3.0; (2)3.0 > ML ≥ 2.0; (3)2.0 > ML ≥ 1.5; and (4) ML = 1.0, 0.5. We shall discuss the location errors by testing synthetics and real data in various situations, comparing with the conventional location method, and comparing the resolution with other seismic networks with different station intervals. The testing results suggest that the machine learning method is capable of meeting the standard of Oklahoma regulations for reporting events of M_L_ ≥ 2.0 in real time to guide the action of local operators in the shale gas production field.

## Results

### Data

The sharp increase in the occurrence frequency of small- to moderate-sized earthquakes in Oklahoma, USA, since 2009 has drawn elevated concerns regarding the potential for earthquake hazards in this area^3,4^. Many studies have shown that the sharp increase in seismicity in Oklahoma is principally caused by the large-scale injection of saltwater into the Arbuckle group^35,36^. To monitor the induced seismicity in Oklahoma, the temporary Nanometrics Research Network consisting of 30 broadband seismic stations operated by Nanometrics Seismological Instruments was deployed in this region from 10 June 2013 to 31 March 2016. The seismic network covers an area of approximately 320 km × 270 km in Oklahoma. The minimum station interval varies from 14 to 30 km, and the signals are recorded from 0.1 Hz to 30 Hz on all three components. A bandpass filter of 2.0–8.0 Hz is applied to process all of the data for our application.

We selected 1,013 events with magnitudes ranging from M_L_ 3.0 to M_L_ 4.9 as cataloged by the U.S. Geological Survey (USGS) along with five times of the events augmented as additional data for accounting for smaller events to train the neural network. The augmentation approach will be introduced in the following. Each event is in a 90 s time window as a continuous record. The starting time of the window for each event is the first break point of the event at the very first recording station. To account for uncertainty of the starting time, we add another training set of the same 1,013 events by applying an arbitrary shift (<10 s) to the starting time. To simulate the real situation, we use the early events as training samples (from 10 June 2013 to 6 November 2015) and the latest events as testing samples (from 7 November 2015 to 31 March 2016). In a later section, different numbers of events in training and testing groups are also tested to study the performance of the neural network.

Current catalog for earthquakes in Oklahoma mainly documents events of M_L_ ≥ 2.5 along with a small number of smaller events (2.5 > M_L_ ≥ 1.5). However, lowering the event magnitude threshold for detection and location to below M_L_ 3.0 is critical for following regulations in Oklahoma. We found 200 events of 3.0 > M_L_ ≥ 2.0 and 53 events of 2.0 > M_L_ ≥ 1.5 in the catalog, and chose them as testing events. But the system needs training events for this magnitude range as well. Therefore, one of our efforts is to construct additional training set for locating and testing smaller events with magnitude below M_L_ 3.0. Taking the 1,013 training events (M_L_ ≥ 3.0) as base data, we scale down the amplitudes of these events to the magnitude randomly ranging from M_L_ 0.5 to M_L_ 3.0 according to the Richter scale for earthquake magnitude but adding with the normal level of noise recorded at the corresponding stations^37^. This approach is more effective than directly using smaller events, since the ground truth of smaller events is subject to larger uncertainty due to lower signal-to-noise ratio. This process is regarded as data augmentation in deep learning, creating new training dataset from base data by making minor alterations. We also repeat the above process for creating testing data in the lower range of 1.5 ≥ M_L_ ≥ 0.5 by scaling the 200 moderate size of testing events (M_L_ ≥ 3.0) and make up missing events in this lower range in the catalog. With these efforts, we should be able to establish capability of the system to locate events in a broad magnitude range of M_L_ ≥ 0.5.

To further test the system and understand its reliability, we also generate synthetic seismograms using the source information of 870 training events and 200 testing events. We demonstrate the advantage of applying the machine learning method over the conventional grid search method for dealing with small events. The testing procedure and results are provided in the Supplementary Information. The system is capable to detect interfered events that are not in the interest zone or a record of just random noise. Examples are presented in the Supplementary Information as well.

### 3D location image

**Figure 1:**
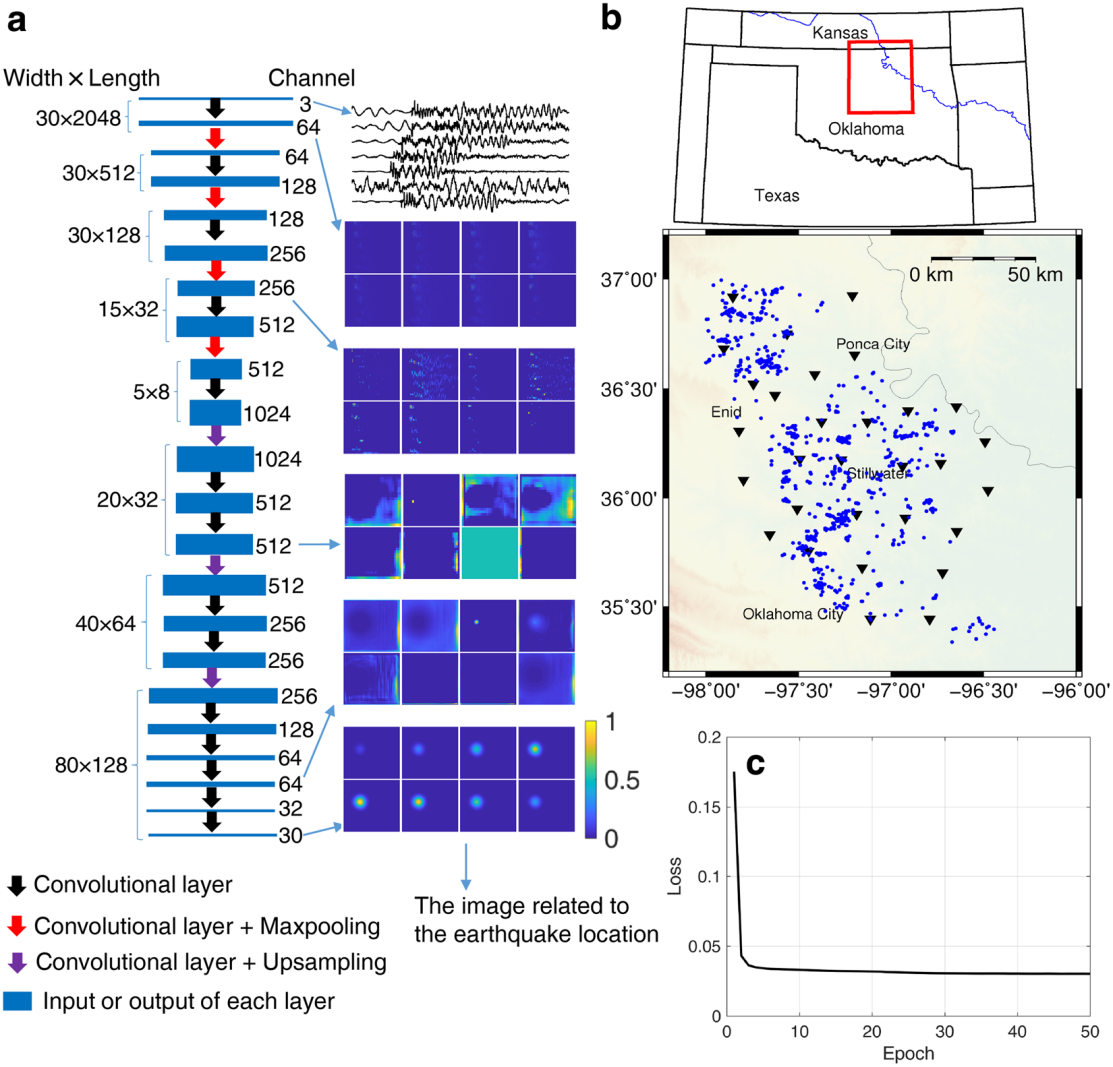
The neural network architecture and network training. (**a**) The sizes of the input and output data are labeled on the left side of the network architecture, and the depth (channel) of the data is labeled to the right. The images of eight selected channels from some of the layers are displayed. (**b**) The red box marks the region of interest, and 1,013 cataloged events (M_L_ ≥ 3.0) (blue dots) are selected for the training set. (**c**) The loss curve during the training is displayed.

In machine learning problems, we need to pair the input data and the output results in a quantitative manner. Due to the underlying physics, there is a nonlinear relationship between the seismogram data and event location parameters in an earthquake location problem. Accordingly, instead of generating a single class label for the earthquake location, our FCN model outputs a 3D image volume that represents the probability of the event location in the subsurface, as shown in the bottom plot of Fig. 1a. The point within the image with the largest magnitude marks the most likely event location. The details of the network architecture illustrated in Fig. 1a will be elaborated in the section of Methods. Through numerical studies with a grid search method to calculate the misfit of the arrival times of an event in the subsurface, we find that the distribution of the misfit somewhat reflects a Gaussian probability for the event location, where the minimum misfit corresponds to the most likely location. Therefore, we represent the ground truth of the event location by a 3D Gaussian function, the peak point of which marks the event location, and the peak value is 1.0. Testing examples with real data reveal that the output may not maintain the shape of a Gaussian function, and the peak value may vary depending on the uncertainty in the result. As shown in Supplementary Information, the peak value will be significantly lower if the true event location of the testing data is outside the 3D volume (see Supplementary Fig. S7). Moreover, the result is expected to be more accurate for higher peak values. Therefore, we are able to eliminate false results using a preset threshold of the probability value. False results may include events outside the interest zone, random noise, or waveform data that do not significantly resemble any event in the training dataset (see Supplementary Fig. S8).

To monitor induced seismicity in Oklahoma, the volume range of our output is constrained by our zone of interest bounded by the latitude range from 34.975° to 37.493°, the longitude range from −98.405° to −95.527°, and the depth range from 0 km to 12 km. As designed in our network, the output volume is 80 × 128 × 30 grids, representing a study area with dimensions of 2.518° (Latitude) × 2.878° (Longitude) × 12 km or 280 km × 259 km × 12 km. This means that the grid spacing in the 3D pixel volume is 0.0315° in latitude (3.5 km), 0.0225° in longitude (2.0 km), and 0.4 km in depth. The number of input and output grids is coupled with the number of layers in the network structure. Synthetic tests show that slightly different grid size in latitude and longitude in this study produces acceptable resolution in both dimensions (see Supplementary). The ground truth is obtained from the USGS earthquake catalog. To train the network, we utilize 200 epochs with a batch size of 4, and apply 10% of the training data as the validation set in the training phase. Fig. 1b,c show the event epicenters of the 1,013 training samples and the convergence of the loss function, which approaches zero after 10 epochs. Consequently, the trained model with the smallest validation loss is ready to predict the locations for the new data with arbitrary starting time.

### Testing with relatively large events (M_L_ ≥ 3.0)

**Figure 2:**
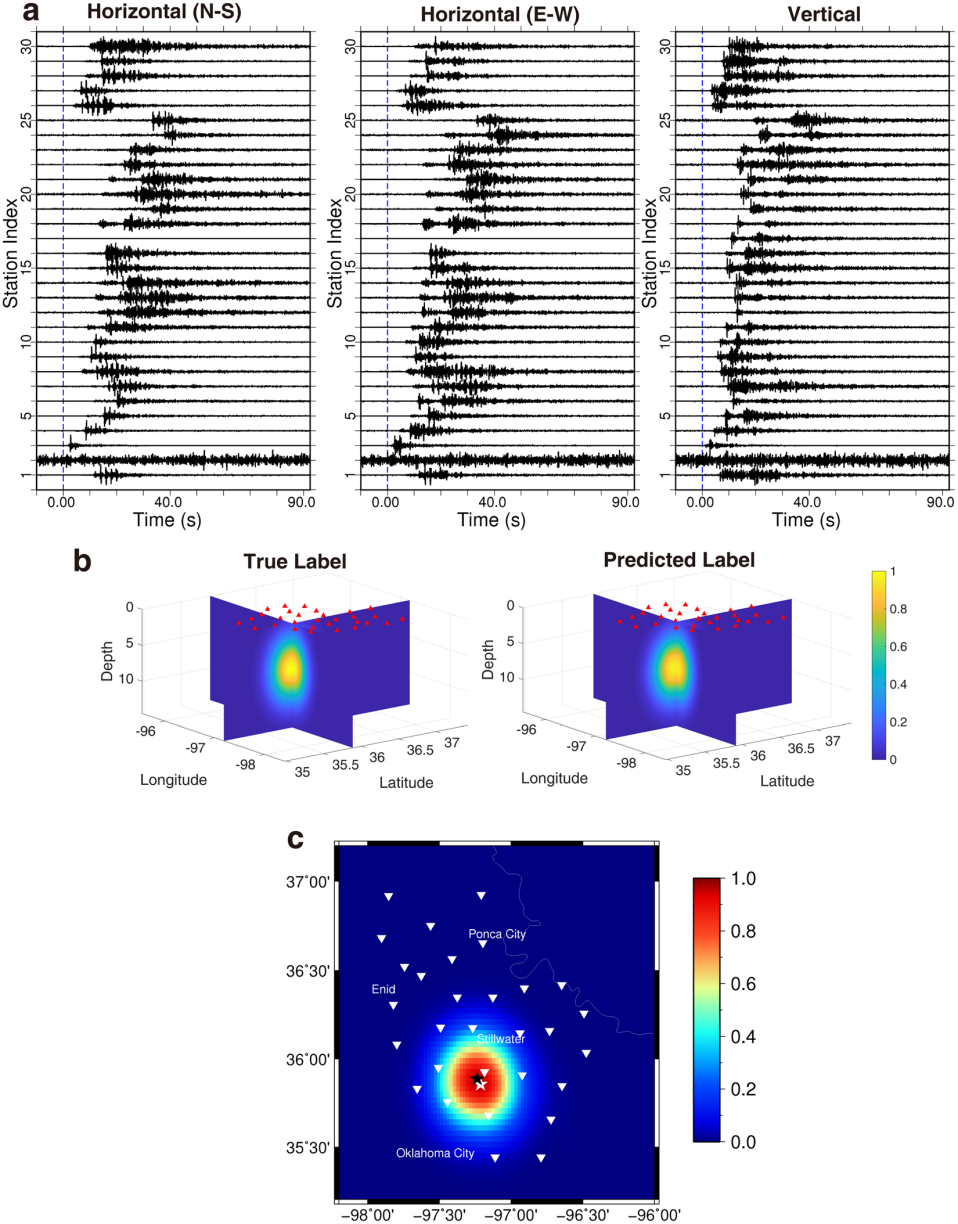
The prediction results for a testing event (M_L_ 3.0). (**a**) The three components of the input waveform for an earthquake on 5 March 2016. (**b**) The true and predicted labels; the red triangles for seismic stations. (**c**) The true and predicted epicenters; the white triangles for seismic stations; the black and white stars for the predicted and true epicenters, respectively; the color image for the location probability in panel c extracted from the 3D volume in panel b.

**Figure 3:**
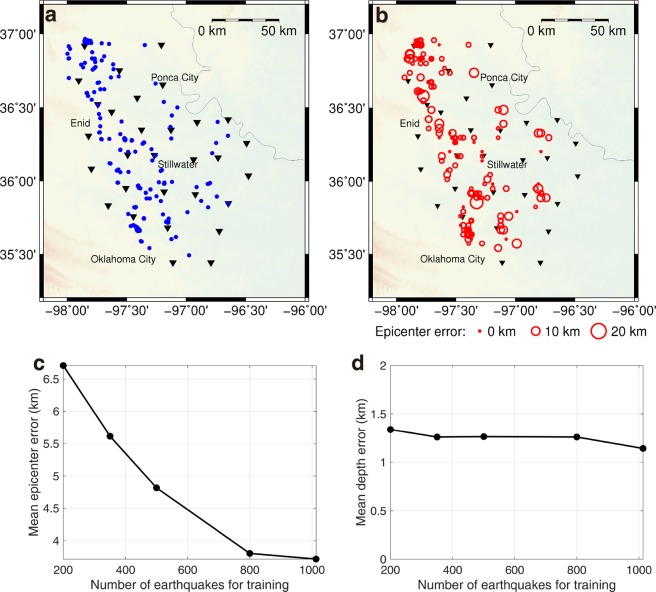
The prediction results for 200 testing events (M_L_ ≥ 3.0). (**a**) The true epicenters of the 200 testing earthquakes (blue dots). (**b**) The predicted epicenters and errors of the 200 earthquakes in (**a**), and the mean epicenter error is 3.7 km. (**c**) The mean epicenter error versus the number of training events employed in the FCN. (**d**) The mean depth error versus the number of training events employed in the FCN.

To assess the location performance of our deep learning algorithm, we first test the 200 relatively large events (M_L_ ≥ 3.0) in Oklahoma with the FCN model. The network takes in three-component data recorded at 30 stations in the form of the RGB color model and produces an output consisting of a 3D location image for each event. In the computer image recognition problem, the input of a color image is represented by the intensity values of the image in three primary colors: red, green, and blue. We correspond our three components of earthquake data to the three primary colors, and convert 3 C seismograms to a color image. Figure 2 shows a testing example with an event that occurred on 5 March 2016, with a magnitude of M_L_ 3.0. The input data are displayed in Fig. 2a, and the predicted 3D location image is shown in Fig. 2b. The value of the peak point is 0.9, and the predicted location is approximately 4.0 km away from the catalog location marked by the white star (Fig. 2c). In this example, the instrumentation of station 14 and 29 might incorrectly function. However, the results are not nearly affected. This is one of the advantages using data from a network as opposed to a single station. It also demonstrates the benefits of applying a deep learning method to work on the patterns in data rather than the precise amplitude values. Figure 3 presents the location results for all of the 200 testing events; the ground truth is illustrated in Fig. 3a, and the testing results are provided in Fig. 3b, in which the size of red circle denotes the epicenter error. The ground truth of these 200 testing events is also obtained from the earthquake catalog produced by conventional processing using time picks. The predicted results suggest that the mean epicenter error is about 3.7 km, and the mean depth error is about 1.1 km. However, these numbers may not reflect the true prediction errors since the calculation uses catalog locations as ground truth with expected errors. The synthetic test in the Supplementary Information suggests that the true prediction error should be only about half of the calculated error referenced to the catalog locations with this system, *i.e*., about 1.9 km for the mean epicenter error in this case.

We further evaluate the performance of the FCN model with a number of different training samples. We use the same 200 testing events to calculate the mean errors of the predicted locations for the FCN model trained with a different number of samples in base data. As shown in Fig. 3c, the epicenter errors decrease with an increase in the number of training samples. The depth errors are generally small over the different number of training samples (Fig. 3d); this may be because most of the training events are in the depth range from 4.0 to 7.0 km in Oklahoma. Therefore, in this study, we primarily focus on the epicenter error in prediction. With approximately 1,000 training events, the location errors seem acceptable and the error curves on Fig. 3c,d suggest more training samples may continue to improve the results. A training set with about 1,000 samples is considered a very small amount of training data in deep learning applications. If we apply the CNN classification method to solve the earthquake location problem with a similar resolution and take each possible location pixel as a class, several hundreds of thousands of classes are needed, and thus, an enormous number of samples would be required to train the network for such a large-scale classification problem. In an image classification example with 1,000 classes, approximately 1.2 million images are required for training^38^.

### Testing with small events (3.0 > M ≥ 0.5)

It is important to detect and locate smaller events less than M_L_ 3.0 for monitoring induced earthquakes. As stated before, Oklahoma regulators require operators to take action if any event of M_L_ 2.0 or above occurs, and operators must be capable of detecting such small events by themselves. In general, the occurrence rate of small events (M_L_ < 3.0) should be much higher than large events (M_L_ ≥ 3.0) in induced seismicity. (3) However, there are not many small events of M_L_ ≤ 2.5 in the USGS catalog for this particular network during its operating periods. Existing methods for processing small events require substantial human efforts. As mentioned before, we found 200 events of 3.0 > M_L_ ≥ 2.0 and 53 events of 2.0 > M_L_ ≥ 1.5 in the catalog, and chose them as testing events. As described in Data section, we then scaled down the amplitudes of 200 relatively large events (M_L_ ≥ 3.0) to the magnitudes M_L_ = 1.5, 1.0, and 0.5, respectively, and created smaller testing events.

**Figure 4:**
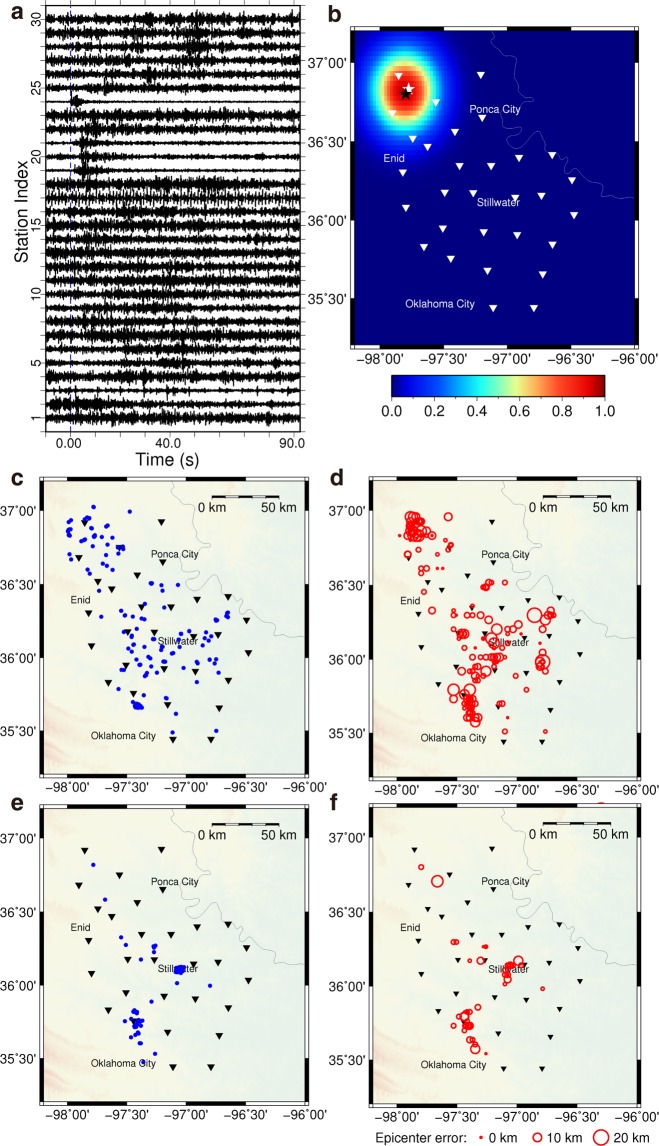
The prediction results for small earthquakes (3.0 > M_L_ ≥ 1.5) in the catalog. (**a**) Z component of the input waveform from an earthquake (M_L_ 1.5) on 30 November 2015. (**b**) The probability distribution of predicted location for the earthquake (M_L_ 1.5) on 30 November 2015; the white triangles for seismic stations; the black and white stars for the predicted and true locations. The color image for the probability of the predicted location. (**c**) The true epicenters of 200 earthquakes (3.0 > M_L_ ≥ 2.0) in the catalog from 29 December 2015 to 17 February 2016. (**d**) The predicted epicenters for the 200 earthquakes in (**c**), and the mean error is 5.3 km. (**e**) The true epicenters of 53 earthquakes (2.0 > M_L_ > 1.5) in the catalog from 30 June 2013 to 9 March 2016. (**f**) The predicted epicenters of the 53 earthquakes in (**e**), and the mean error is 4.1 km.

The vertical components of a small event of M_L_ 1.5 over 30 stations on 30 November 2015 are displayed in Fig. 4a. Noise is substantial, and two stations (3 & 17) might malfunction. Its predicted epicenter with the highest probability in the Earth is marked by the black star in Fig. 4b, and the white star is the location from the catalog. The epicenter error is 4.0 km in this case. However, the epicenter errors for events of M_L_ 1.5 could vary greatly depending on data quality and relative event location within the network, which will be further tested in the following. The catalog locations of 53 testing events of 2.0 > M_L_ ≥ 1.5 are plotted in Fig. 4e, and the testing results along with prediction errors are shown in Fig. 4f. The mean epicenter error of the 53 events is 4.1 km. On the other hand, for the slightly larger events of 3.0 > M_L_ ≥ 2.0 (Fig. 4c), the mean epicenter error of the 200 events is 5.3 km (Fig. 4d). Slightly larger magnitude does not necessarily lead to smaller prediction error. Because of different event locations, these results may not be directly comparable, but indicating an approximate range of 3.7-5.3 km in the prediction error. As discussed in synthetic testing, considering the location errors in the catalog, this actual error range may be smaller.

**Figure 5:**
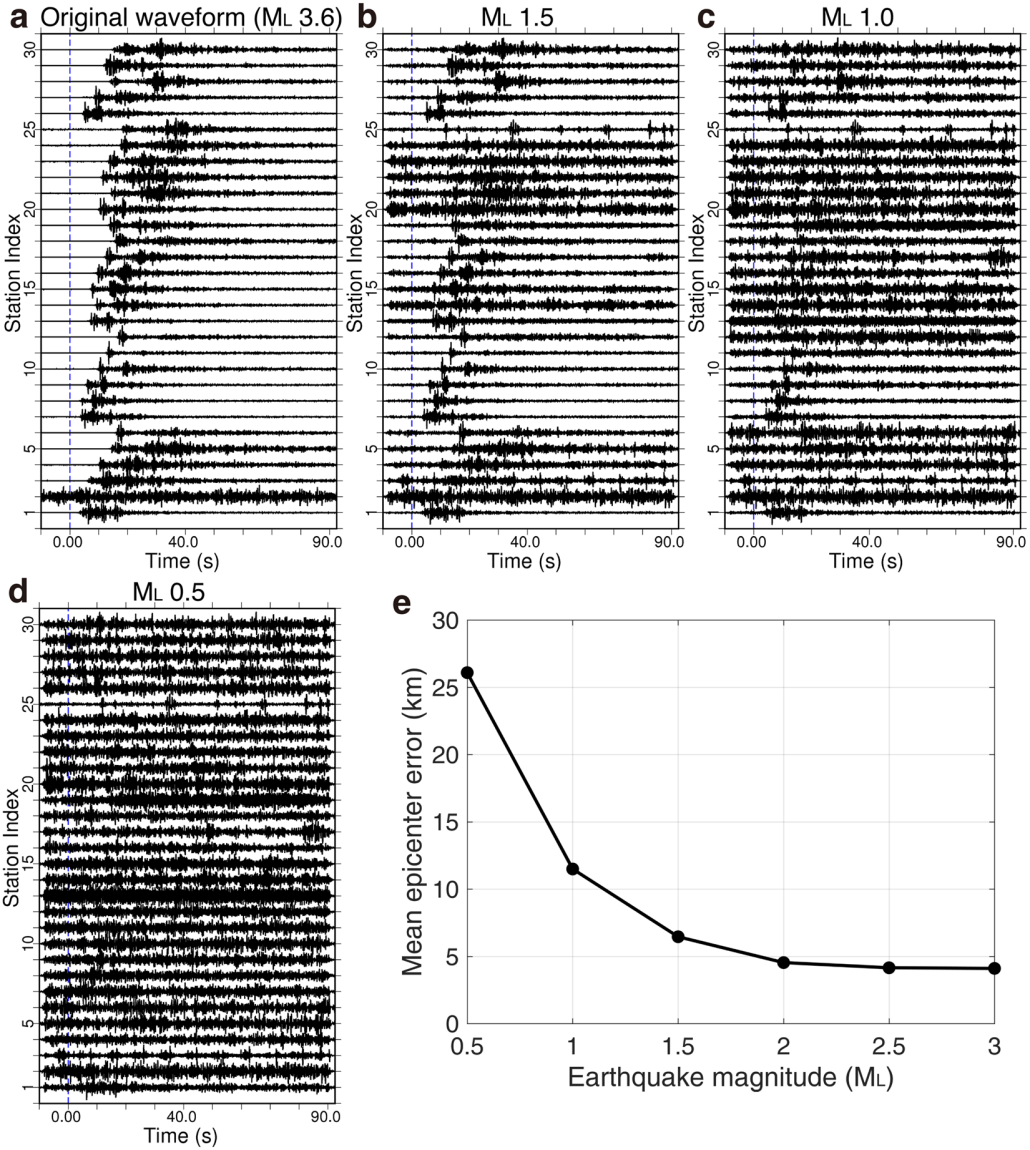
Simulating smaller events by scaling amplitude and testing prediction errors. (**a**) Vertical seismograms of an event of M_L_ = 3.6. (**b**) A simulated event of M_L_ = 1.5. (**c**) A simulated event of M_L_ = 1.0. (**d**) A simulated event of M_L_ = 0.5. (**e**) A plot of event magnitude versus epicenter error is produced through repeated prediction testing for 200 simulated events at each selected magnitude.

Testing smaller events of 1.5 ≥ M_L_ ≥ 0.5 is more challenging for predicting accurate results. In general, smaller events present weaker signals and suffer from relatively larger noise impact. The subsurface environments in Oklahoma are also highly noisy due to over 200,000 active wells operating in the state. As an example, the vertical component of an earthquake of M_L_ 3.6 is displayed in Fig. 5a, and scaled events of M_L_ 1.5, 1.0, and 0.5 from the event of M_L_ 3.6 are also displayed in Fig. 5b–d. The same level of regular noise is added to the simulated events, thus events with smaller magnitude show larger noise. By scaling down all of the 200 testing events (M_L_ ≥ 3.0) to a lower magnitude each time and repeatedly testing the predictions, we generate a plot of epicenter error versus event magnitude (Fig. 5e). For the simulated events with M_L_ 1.5, 1.0, and 0.5, the epicenter error of the prediction is 6.4, 11.5, and 26.1 km, respectively. These results suggest predicting smaller events of 1.5 > M_L_ ≥ 0.5 with data from this network in Oklahoma is difficult. A denser station network is needed to record higher quality of data within shorter distances.

## Discussion

We propose a fully convolution network to predict the hypocentral locations of small earthquakes in the form of 3D probabilistic distribution images and apply the approach to monitor induced seismicity in Oklahoma. The testing results suggest that the system is capable of locating small events of M_L_ ≥ 2.0 with a mean epicenter error of 4-6 km, meeting the demand of Oklahoma regulations for monitoring induced earthquakes. Considering the station interval in the seismic network in this study varies from 14 to 30 km, we believe these results are fairly reasonable^39^. To further improve the results and reduce the location errors, we must install denser seismic stations in the area. Nevertheless, this study demonstrates that the machine learning approach is promising to deal with a large amount of data in a real-time fashion, and to offer a robust solution for monitoring small induced earthquakes, which has been a challenging task even for human processing.

The limitation of the method is the accuracy of ground truth and the coverage of the training set. The ground truth from catalog may include errors. Improvement on the ground truth could be made by further refining time picks manually and applying relative location methods such as the double difference approach to minimize the influence of velocity heterogeneities^11^. The number of training samples is important, but the coverage of the training samples in the interest zone is also essential. We observed that the large location errors are mainly in the areas without many training samples. The deep learning method requires training data, which means a newly installed network cannot apply the method with cataloged events. For a monitoring system that has been already trained for a seismic network, a couple of malfunction traces do not seem affecting results much (see Fig. 2), but the system does not allow adding new stations arbitrarily. A potential effort is to apply synthetics as a training set, which warrants further study.

The study area (280 km × 259 km) is relatively large with 1,013 training samples. Both the size of the study area and the number of training samples affect the accuracy of the predicted locations. Our extensive tests suggest that finer grids in the output image do not necessarily help produce higher resolution for location due to limited training samples. However, the induced earthquakes in Oklahoma mostly occur in a small depth range of 2-8 km. Testing the neural network with a small depth interval seems producing accurate depth results, but the ground truth of depth from the training data is often subjective due to limited physical constraints in data. Therefore, the depth resolution and accuracy is only relative to the ground truth of the training data.

In Supplementary Information, we demonstrate the effects of a number of factors in our approach with synthetics and real data, including noise, uncertainty in the initial arrival time, partial data, and the radius of Gaussian distribution. We also tested the network with interfered events and noise, and compared the deep learning method with the conventional grid search method. The deep learning method does not require picking the first arrivals, thus, it can handle small earthquakes with low signal-to-noise ratio. It utilizes both phase and amplitude information from data in training, validation, and testing.

Nevertheless, this study demonstrates that the machine learning approach is promising to deal with a large amount of data in a real-time fashion, and to offer a robust solution for monitoring massive small induced earthquakes, which has been a challenging task even for human processing. The use of the machine learning approach for event location and many other applications in seismology simply develops a new path that integrates and connects the data and knowledge obtained between the present and the deep past, to gain new insights instantly.

In this application, there are 21 layers in the network architecture, which means the number of trainable parameters is large and the network model may be over-parameterized. However, the over-parametrization is widely adopted in deep learning because it can help the generalization for neural networks as shown in recent studies^40,41^. The mapping from waveforms to the location image is complex, therefore, the prediction function should be represented by a deep neural network. On the other hand, for the large size networks, the global optima are in a sense ubiquitous when training the networks, and the overfitting problem caused by the large size networks can be prevented by the strong regularizations. The use of machine learning in solid Earth geosciences is growing rapidly, but is still in its early stages^42^. In this study, we intend to solve the small earthquake location problem by applying the fully convolutional network, which directly benefits implementing the traffic light system for monitoring induced earthquakes. The approach could be further applied to help make new discoveries in seismology by processing large datasets associated with small earthquakes.

## Methods

This study assumes that seismic events have been already detected, and the input to our neural network is an event within a selected time window. Event detection is a unique problem in seismological studies, which has been well studied^12–21^. In practice, detection continues to calculate in real time, but event location is executed only when selected data is provided, an approach similar to all other automated methods. Our location method constitutes a fully convolutional network (FCN) that takes in a window of three-component waveform data from multiple stations as volumetric input and predicts the earthquake location with a 3D image as the output. We propose the use of a 3D Gaussian distribution in the subsurface to delineate the probability distribution of an earthquake location, where each pixel represents a label with a probabilistic value. The peak position in the output volume represents the most likely earthquake location, and the magnitude of the peak value represents the probability of the result.

For each training event, we label the input data with the following Gaussian distribution:

1 $$\{\begin{array}{c}f(x,y,z)=\exp \{-[{(x-{x}_{0})}^{2}+{(y-{y}_{0})}^{2}+{(z-{z}_{0})}^{2}]/r\}\\ x\in {R}_{x},y\in {R}_{y},z\in {R}_{z}\end{array}$$

where $({x}_{0},{y}_{0},{z}_{0})$ denotes the location parameters of the earthquake, the ground truth is obtained from the U.S. Geological Survey earthquake catalog, ${R}_{x}$, ${R}_{y}$, and ${R}_{z}$ are the dimensional limits of the 3D zone of interest, and $r$ is the radius of the Gaussian function.

### Network architecture

Our network is mainly composed of convolutional layers, and the fully connected layer is abandoned in comparison with the convolutional neural network (CNN) classification method. The fully connected layer is commonly used as the final layer to output a vector of classification probabilities in image classification problems^38^. However, for an earthquake location problem, thousands of classes are required if each location pixel is set as a class, and such a large-scale classification problem may require an enormous number of training samples to achieve an accep` accuracy; unfortunately, the number of cataloged earthquakes is limited. Therefore, similar to the methods used in image segmentation^32^, we choose to directly utilize the final convolutional layer to output a 3D volume of pixels representing the probability of an earthquake location.

The convolutional layer in the network architecture (Fig. 1a) is formulated as follows:

2 $${y}_{ijc}^{l}=\sigma (\mathop{\sum }\limits_{a=1}^{m}\mathop{\sum }\limits_{b=1}^{n}\mathop{\sum }\limits_{c^{\prime} =1}^{{C}^{l}}{y}_{(i+a)(i+b)c^{\prime} }^{l-1}{w}_{abc^{\prime} }^{c})$$

where ${y}^{l}$ is the output of the layer $l$; $w$ contains the weights for the filters in the current convolution layer; the output and input channels are indexed with $c$ and $c^{\prime}$, respectively; the number of channels in layer $l$ is *C*^*l*^; the kernel size of the filter is $m\times n$; and $\sigma$ is the nonlinear activation function. The input and output of the convolutional layer in Eq. (2) are both 3D arrays with dimensions of width, length and channel, and each channel of the layer output is obtained by convolving the channels of the previous layer with a bank of 2D filters applied in the width and length directions, as shown in Eq. (2) We utilize the zero-padded convolutional layer in the whole network; therefore, the width and length of the input are the same as those of the output. We utilize 21 convolutional layers in the neural network, as shown in Fig. 1a. The input of the network contains three-component waveform data normalized with the maximum amplitude, and each component corresponds to a channel of a color image simulating one of the RGB colors. The total number of seismic stations is 30, and the number of time samples of an event extracted from a data trace is 2048. Therefore, the input data are represented by a 3D volume (184,320 points) with dimensions of 2048 (time samples) by 30 (stations) by 3 (components). We set the kernel size $m\times n$ of all convolutional layers to be 3 × 3. The number of channels of features is increased from 3 to 1024 and then decreased from 1024 to 30. The output feature size of a convolutional layer is also determined by the number of channels in the current layer, and the weights $w$ are also related to the number of channels of the input feature according to Eq. (2) We utilize the rectified linear unit (ReLU) activation function in each layer, but the sigmoid function is utilized in the final convolution layer to output the final Gaussian distribution image.

In our application, we also apply downsampling (maxpooling) and upsampling to the output of some of the intermediate convolution layers. The maxpooling layer is utilized to extract useful information from the waveform data. However, the input size (30 × 2048 × 3) is very different from the output size (80 × 128 × 30) in our application. The maxpooling and upsampling operations are also able to adjust the width and length of the features in the intermediate layers of the network, as shown in Fig. 1a. For example, we set the pooling size to be^1,4^ in the first maxpooling operation for the output of the 3^rd^ convolutional layer, the width remains unchanged, and the length is decreased 4 times. The width and length of the features become 5 × 8 after the four maxpooling operations with pooling sizes of, (1, 4), (1, 4), (2, 4) and (3, 4) To obtain the final location image, we utilize upsampling to increase the size of the features; the sizes for the three upsampling operations are, (4, 2), (2, 2) and, (2, 2) which means we repeat the rows and columns of the data by the two values of the size, respectively. Finally, the width and length of the features are increased to 80 × 128 after the three upsampling operations.

We output the 2D features by selecting eight channels from the 3D outputs in some of the layers to show how the waveform is transformed into the location image through each layer (Fig. 1a). Because the maxpooling layer is used to extract the features that are sensitive to the earthquake location, the features from the layers before the final maxpooling layer are similar to the input. However, the features are gradually transformed into the final Gaussian image as the layers become deeper.

### Objective function of the training

We utilize a set of three-component waveform data from 30 seismic stations labeled with 3D probabilistic location images to train the network, and we adopt the binary cross-entropy loss function as follows:

3 $$\Psi =\frac{1}{N}\mathop{\sum }\limits_{k=1}^{N}\sum _{d\in D}{p}_{d}^{k}\,\log ({q}_{d}^{k})\,+\,(1-{p}_{d}^{k})\log (1-{q}_{d}^{k})$$

where $p$ and $q$ are the predicted and true location image labels in this study; $N$ is the number of training samples; and $D$ is the assemblage of grid nodes in the location image. Because both the waveform data and the location image label require a substantial amount of memory for training, we minimize the loss function $\Psi$ using a batched stochastic gradient descent algorithm. The samples are shuffled prior to training, and then we divide the samples into several batches. At each training step, we feed the neural network a batch of samples and minimize the loss function to obtain the updated FCN model. This process is repeated until all samples are fed into the neural network, and then the current epoch is finished. We utilize 200 epochs to train the neural network with about four hours for 1,013 samples. The prediction is fast, and the total time use for the 200 earthquakes (200 waveform windows) are 3.017 s by GPU computation (GeForce GTX 1080), which means one hundredth of a second per event.

We perform the tests based on TensorFlow^43^, and the Adam algorithm is utilized to optimize the loss function for each batch at each epoch^44^. The learning rate is set to 10^−4^, and the other parameters are set to the default values recommended by the authors of the Adam algorithm. We also utilize two dropout layers after the 8th and 10th convolutional layers respectively to regularize the training to avoid overfitting the data, and the rates for the two dropout layers are 0.5. We also utilize 10% of the samples used for training as validation set to select the neural network models and prevent the overfitting problem.

## Supplementary information

Supplementary information

## Data Availability

The data used in this study can be requested from IRIS website: http://ds.iris.edu/ds/. The information of the seismic network is described by the website: http://www.fdsn.org/networks/detail/NX. The ground truth of the earthquake events are obtained from USGS website: http://earthquake.usgs.gov/earthquakes/search/ We open the source codes, please contact corresponding author.
