# Supplementary material for: Locating induced earthquakes with a network of seismic stations in Oklahoma via a deep learning method
Source: Sci Rep. 2020 Feb 6;10:1941. doi: 10.1038/s41598-020-58908-5 (PMC7005003; doi:10.1038/s41598-020-58908-5)
Supplement: Supplementary file 1 — Supplementary information [file 41598_2020_58908_MOESM1_ESM.docx]

**Locating induced earthquakes with a network of seismic stations in Oklahoma via a deep learning method**

Xiong Zhang^1^, Jie Zhang^1^*, Congcong Yuan^1^, Sen Liu^2^, Zhibo Chen^2^, Weiping Li^2^

^1^School of Earth and Space Sciences, University of Science and Technology of China, Hefei, Anhui 230026, P. R. China.

^2^School of Information Science and Technology, University of Science and Technology of China, Hefei, Anhui 230026, P. R. China.

Supplementary Information

**Testing the deep learning method with synthetics**

In dealing with real data, the ground truth information of earthquake events is obtained from catalog with expected errors. How does that affect the performance of our system? To answer the question, we apply synthetic data with known ground truth perturbed by errors in location to repeat the above tests. We calculate the synthetic waveforms with the source information of the 870 events for training, and 200 events for testing in the above real data studies for a given 1-D velocity model by applying the generalized reflection and transmission method (*1, 2*). The source focal mechanism solutions of these events are obtained from the source moment tensor inversion of real data. To simulate the real situation in practice, we disturb the true epicenters and depths of the training events with various errors in the training stage, and evaluate the corresponding testing results of the network with known ground truth.

Fig. S1a shows the training events with true location, and Fig. S1b presents the testing results of the 200 events and their epicenter errors relative to the exact ground truth. The mean error of the predicted epicenters is about 2.0 km, and depth error is 0.3 km if the neural network is trained with true locations. This is a system error (error - a), which tells how well the system can handle nonlinearity of the prediction problem and the sparse distribution of training events for this particular case. For the same velocity model, station, and event setting, we also applied a conventional location method, grid search, to locate the testing events using theoretical travel times as input data. The results suggest a mean epicenter error of 1.2 km, and a mean depth error of 1.6 km. Although this test assumes perfect time picks, it suggests a similar system error to the machine learning method. If the ground truth (X, Y, and Z coordinates) of training data is added with random Gaussian errors with the mean error (error - b) of 6.3 km, and 19.0 km, respectively (Fig S1c and S1e), the mean errors of the epicenters (error - c) in the prediction results are 4.9 km and 10.0 km for these 200 synthetic events, respectively (Fig. S1d and S1f).

From these synthetic results, we found that the prediction error (error - c) is generally smaller than the mean location error in training data (error - b), but in the same order. In real data cases, it is reasonable to assume that the location (catalog) errors in both training and testing data are in the same order. Therefore, the maximum of the total calculated errors in our real data testing results should include the system error (error - a), the prediction error due to catalog errors in training data (error - c), and catalog errors in testing data used for accuracy check (error - b). Since the actual prediction error should just include (error - a) and (error - c), and a+c ≈ b, it is reasonable to conclude that the maximum of the true prediction error in our previous real data testing should be just about half of the calculated prediction error (since (a+c+b)/2≈b). We should also point out that these analyses and conclusion are based on the noise-free synthetic waveforms from a 1-D velocity model.

**A comparison between the deep learning method and grid search**

The problem that we intend to address in this study is to locate small earthquakes that are difficult to solve by applying conventional methods. We illustrate the challenge by testing synthetics added with random Gaussian noise. The synthetics that we generated in the section 1 of Supplementary are contaminated by 20% and 50% of Gaussian noise, respectively. The percentage value is measured by the RMS amplitude of noise relative to signal. All of the input and output parameters are the same with those in the last section, without location errors in the training samples. The travel time grid search first applies a STA/LTA method to pick the arrival time (3), which is a common approach for processing large earthquakes. The true 1-D model used for synthetic calculation is applied to calculate a travel time table for the grid search.

Fig. S2a and S2b show the location errors relative to ground truth in both noise levels by applying the deep learning method to process 200 testing events. Fig. S2c and S2d show the corresponding location errors due to the grid search method. Without any picking needed, the deep learning method is able to locate events with average epicenter errors of 2.1 km in the case of 20% Gaussian noise, and 2.4 km in the case of 50% Gaussian noise. The grid search method along with STA/LTA picking produces average epicenter errors of 11.9 and 13.4, respectively.

Fig. S3 illustrates a fundamental reason that the conventional grid search method cannot perform well for locating small events automatically. Fig. S3a and S3b show a synthetic event contaminated by 20% and 30% Gaussian noise, respectively. Fig. S3c and S3d display the STA/LTA calculation result in each case, which is further used for the determination of the first arrival time. The blue points in Fig. S3c mark the true arrival times of P waves. In both cases, it is challenging to extract the first arrival times at far distances accurately. The second example in Fig. S3d with higher noise level is more difficult to pick the true arrivals correctly.

**Locating small events with different window length**

The input data size of the neural network is fixed by$30 \left( \mathrm{stations} \right)\times2048 \left( data samples \right)\times3 (components)$, and the maximum length of the time window is set to be 102.4 s. However, the network does allow using a partial window of data to locate events. Using input data up to 20 s and 30 s with signals and setting the remaining with zeros, we produce the epicenter solutions and calculate errors for the 200 testing events of M_L_ 1.0 and M_L_ 1.5 as shown in Fig. S4. All the parameters in the network are same with the previous tests except for the training data also truncated to 20 s and 30 s accordingly. The results show average RMS errors of 11.9 km and 11.6 km for the earthquakes of M 1.0, and 6.5 km and 6.5 km for the earthquakes of M 1.5. Repeated testing for events of magnitude from M_L_ 0.5 to M_L_ 3.0 with 0.5 as an interval shows close results between two different window lengths except for M_L_ 0.5, for which the errors are separated by over 5 km (Fig. S5).

**Locating earthquakes with progressive window length**

In another effort, we may also start to locate events with an arbitrary time window length of data, and progressively include more data for further improvement on location. Initially maybe only a couple or a few stations record data, and more stations will join and offer data subsequently. To do that, we need to design a training set with signals in arbitrary window lengths. With 1,013 training events, we define new training samples by randomly selecting three windows for each event and setting the remaining portion of data to zero. The lengths of the random windows vary from 5 s to 90 s for each event. This effort augments training samples to 4,052 from 1,013 along with the original samples, and the neural network trained with the augmented training samples is able to predict the locations with progressive time windows. Fig. S6 shows the prediction results for an event of Mw 4.3 with record in three different time windows: 5 s, 10 s, and 20 s. The initial arrival of the seismic event is recorded by 2, 10 and 26 stations progressively, as shown in Fig. S6a, S6b and S6c. In the case of short window of input data, the epicenter should be close to the initially triggered stations and far from the stations which have not received signals, thus, both the triggered and un-triggered stations provide constraints for locating the event. Fig. S7 shows mean errors for the 200 testing samples with the waveforms muted by different time windows. The results are generally improved as more data are included.

We leave 2048 time samples for the input size in consideration of both the real earthquake records and the neural network architectures. However, the real length of the waveform could be arbitrary within 2048 time samples. If we train the neural network with the waveforms aligned to the beginning of the input layer, the truncated waveforms used for prediction should be placed at the same position when there are an event detected. Similar to the image classification for dealing with different size of input images, we should use the same zero-padding rules to handle the input of the training and testing data.

**Preventing false results from interfered events or noise only**

Our deep learning network is designed to monitor induced seismicity in Oklahoma. What will it happen if an event originates from outside the interest zone? To address this concern, we design a test with an earthquake occurring outside of Oklahoma, as shown in Fig. S8. This ML 4.0 event (blue star) occurred at 37.429°, -98.954° on 23 May 2015, and it was approximately 123 km away from the nearest seismic station of the network. Using our FCN model, the event is located within the upper-left corner of Oklahoma. This incorrect location is anticipated because all of the output from our FCN model should consist of events that are located inside the preset 3D volume. However, in this case, the peak value of the output 3D image is only around 0.6, which is much smaller than the peak values for all of the events occurring inside the 3D volume (>0.9), suggesting a very low location probability. Therefore, we can eliminate and prevent false results on the basis of the peak value using a preset threshold.

There is a possibility that event detection fails and a window of random noise is taken as an input for location. However, in this case, noise is significantly different from event training data, which may lead to an extremely low peak value in the probability image. As shown in Fig. S9, an example of noise input shows 0.0023 in the probability image. Therefore, a preset threshold for the peak probability value can help eliminate these abnormal situations and avoid false results.

**Training the neural network with a small portion of samples**

In order to test the generalization performance, we utilize a small portion of samples to train the neural network, then apply a large number of independent testing samples to verify if the neural network is still able to predict locations reasonably well. We first sort the whole samples in the dataset (1,213 earthquakes) randomly, then utilize 500 independent samples to test the neural networks trained with different number of samples. Fig. S10 shows the mean epicenter errors by the neural networks trained with different number of samples. The testing results show that the more training samples, the results are better. If the number of training samples is close to the testing samples, the error is about 5 km.

**The radius parameter of the Gaussian distribution**

The radius of the Gaussian distribution for labeling the location is important for both the convergence of network training and the resolution of the location result. A small radius means that the output matrix of the neural network is sparse, and the non-zero values of the output should be in the small areas around the true location. However, the loss function to be optimized is the similarity measurement between the output and true location image, and a sparse location image is difficult to make the training converging. In order to select a proper radius parameter for the training, we utilize a relatively small training set (500 samples) to try different parameters and then select the parameter yielding the best neural network performance (Fig. S11). We utilize 200 independent testing samples to evaluate the location results predicted by the neural network trained with different radius parameters as shown in Fig. S11. It is difficult to make the training converging by setting a radius parameter too small (e.g. 100), especially when using random values to initialize the weighting of the network, and the output location image tends to be zeroes in the cases by using the values less than 100.0 in our examples. However, large radius of the Gaussian function may decrease the resolution of the location. The location images with large radius parameters are similar to each other for the earthquakes located in similar positions. Although the training is easy to converge by using too large radius, the location errors tend to be relatively large since the resolution decreases.

**The waveform comparison filtered with different frequency ranges for the small earthquake**

To scale down large events and superimpose with noise, we need to ensure that the frequency contents from large events are close enough to those of the small events. Since we apply a bandpass filter of 2-8 Hz to all large events, we need to make sure that actual small events after filtering with the same filter are not altered significantly. We select a small earthquake (M_L_ 1.5) occurred on 30 November 2015 and filter the raw data with frequency ranges of 2-8 Hz and 2-15 Hz respectively as shown in Fig. S12. The comparison shows that the data are well preserved in the frequency of 2-8 Hz (Fig. S12c).


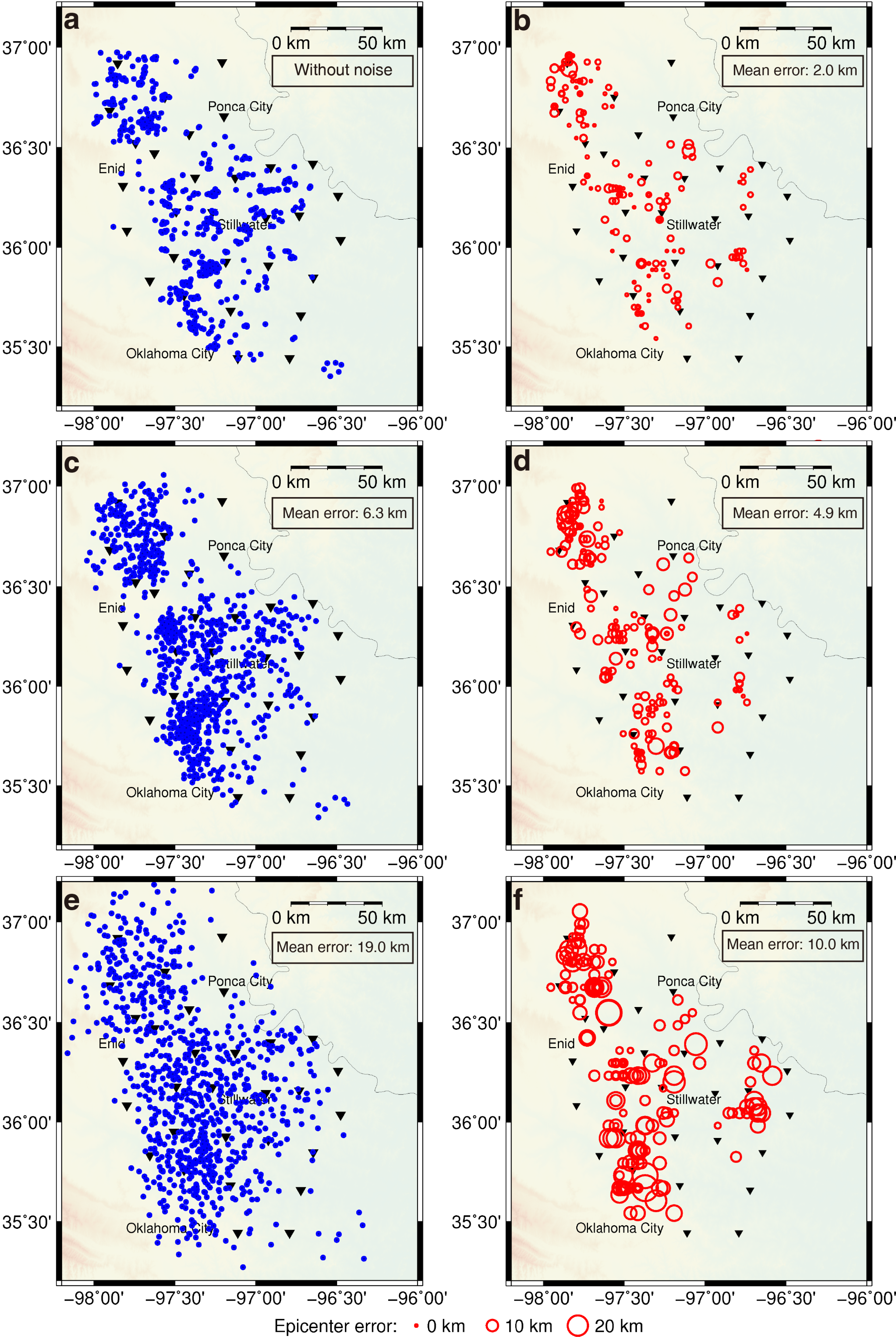


**Fig. S1. The prediction results for synthetic tests.** (a) The true epicenters of the 870 synthetic training events. (b) The predicted epicenters of the 200 testing events by the FCN model trained with the true locations of training events as in (a), and the mean epicenter error is 2.0 km. (c) The epicenters of training events randomly perturbed by a mean location error of 6.3 km. (d) The predicted epicenters of the 200 testing events by the FCN model trained with the perturbed locations of training events as in (c), and the mean epicenter error is 4.9 km. (e) The epicenters of training events randomly perturbed by a mean location error of 19.0 km. (f) The predicted epicenters of the 200 testing events by the FCN model trained with the perturbed locations of training events as in (e), and the mean epicenter error is 10.0 km. The size of the red circles denotes the epicenter error of the prediction.


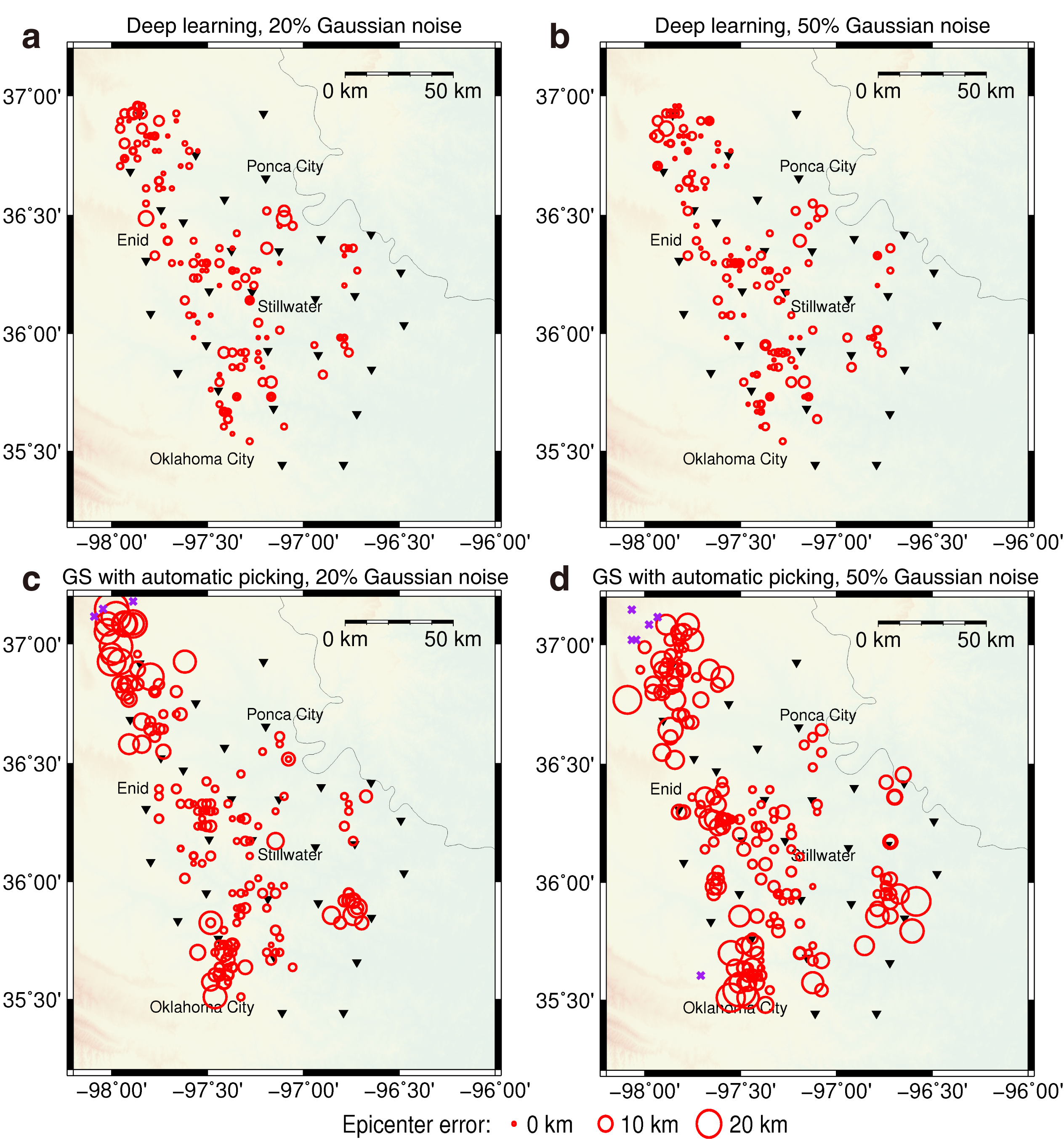


**Fig. S2:** The location results for deep learning method and traveltime time method. (A) The predicted locations for the 200 testing earthquakes contaminated with 20% Gaussian noises. (B) The predicted locations for the 200 testing earthquakes contaminated with 50% Gaussian noises. (C) The location results by traveltime grid search with the automatic phase picking from the waveforms contaminated with 20% Gaussian noises. (D) The location results by traveltime grid search with the automatic phase picking from the waveforms contaminated with 50% Gaussian noises. The purple crosses denote the earthquakes located with errors larger than 30 km.


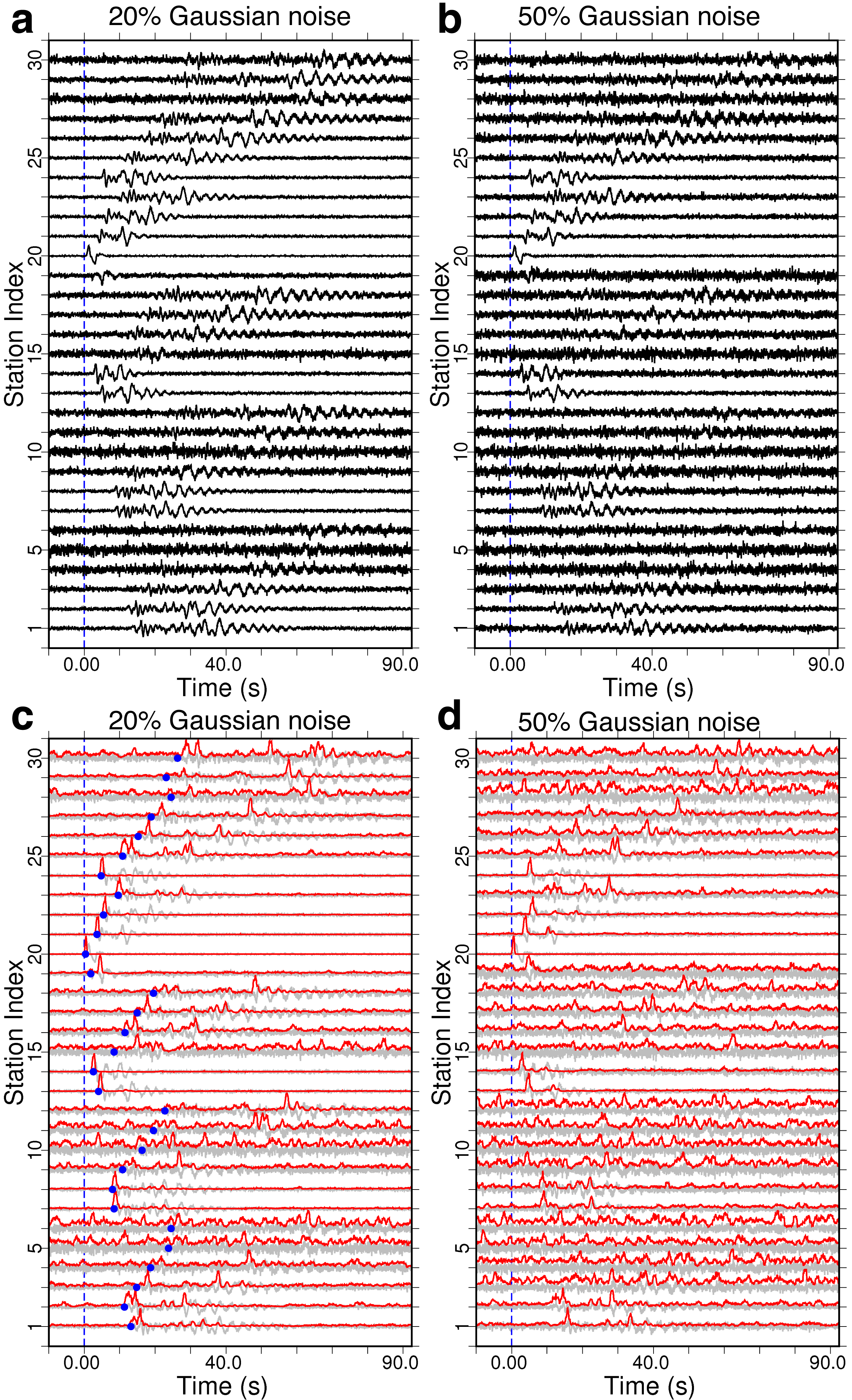


**Fig. S3:** Synthetic waveforms contaminated with Gaussian noise: (a) 20% Gaussian noise, (b) 50% Gaussian noise. The red lines overlaid the waveforms (grey) represent the STA/LTA function for each trace: (c) for data with 20% Gaussian noise; (d) for data with 50% Gaussian noise. The blue dots are the ground truth of arrival times, which are the manual picks from noise-free waveforms.


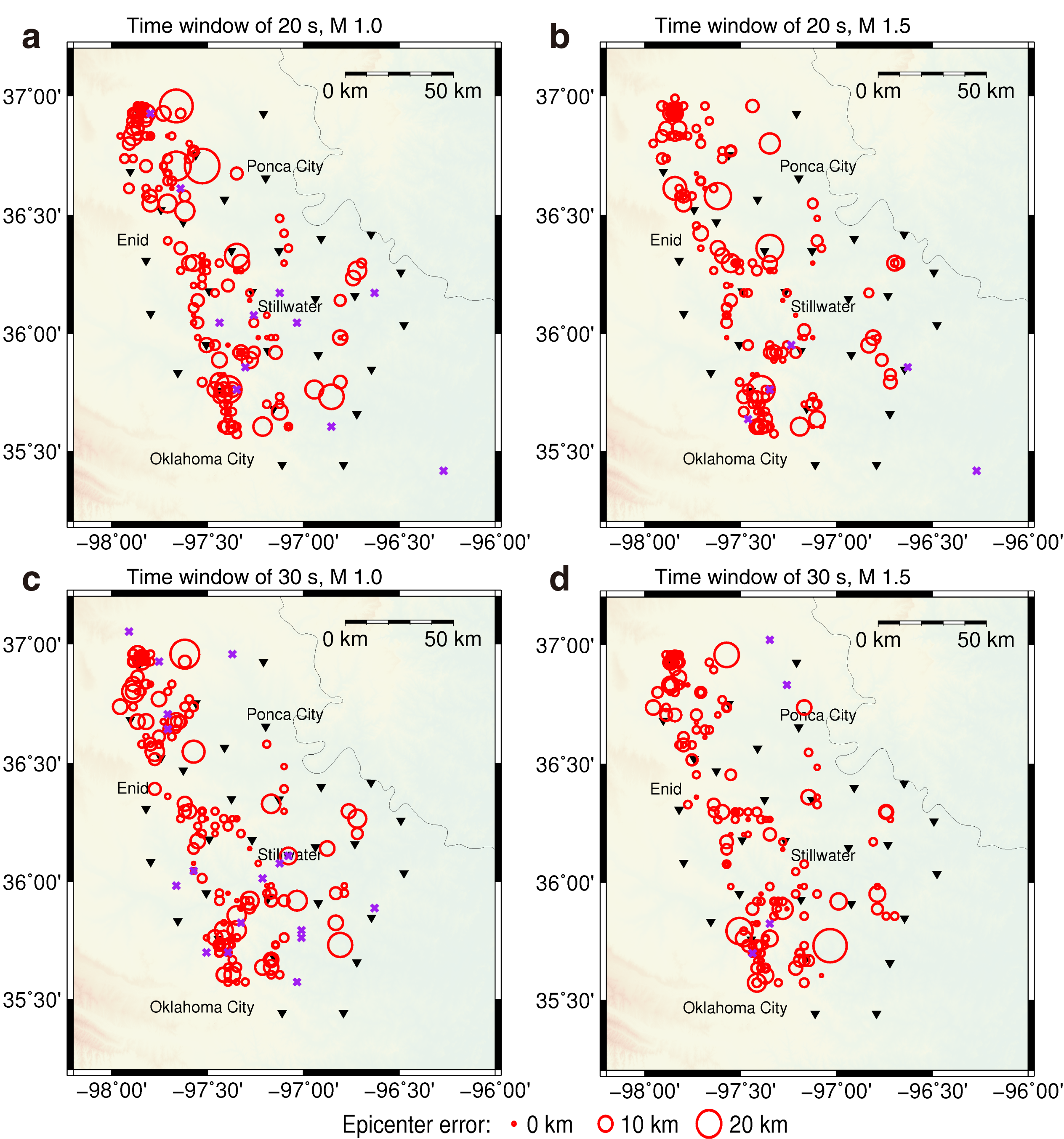


**Fig. S4:** The predicted locations for the small earthquakes with short time windows. (a) The location results for the 200 testing earthquakes of M 1.0 with waveform data of 20 s. (b) The location results for the 200 testing earthquakes of M 1.5 with waveform data of 20 s. (c) The location results for the 200 testing earthquakes of M 1.0 with waveform data of 30 s. (d) The location results for the 200 testing earthquakes of M 1.5 with waveform data of 30 s. The purple crosses denote the earthquakes located with errors larger than 30 km.


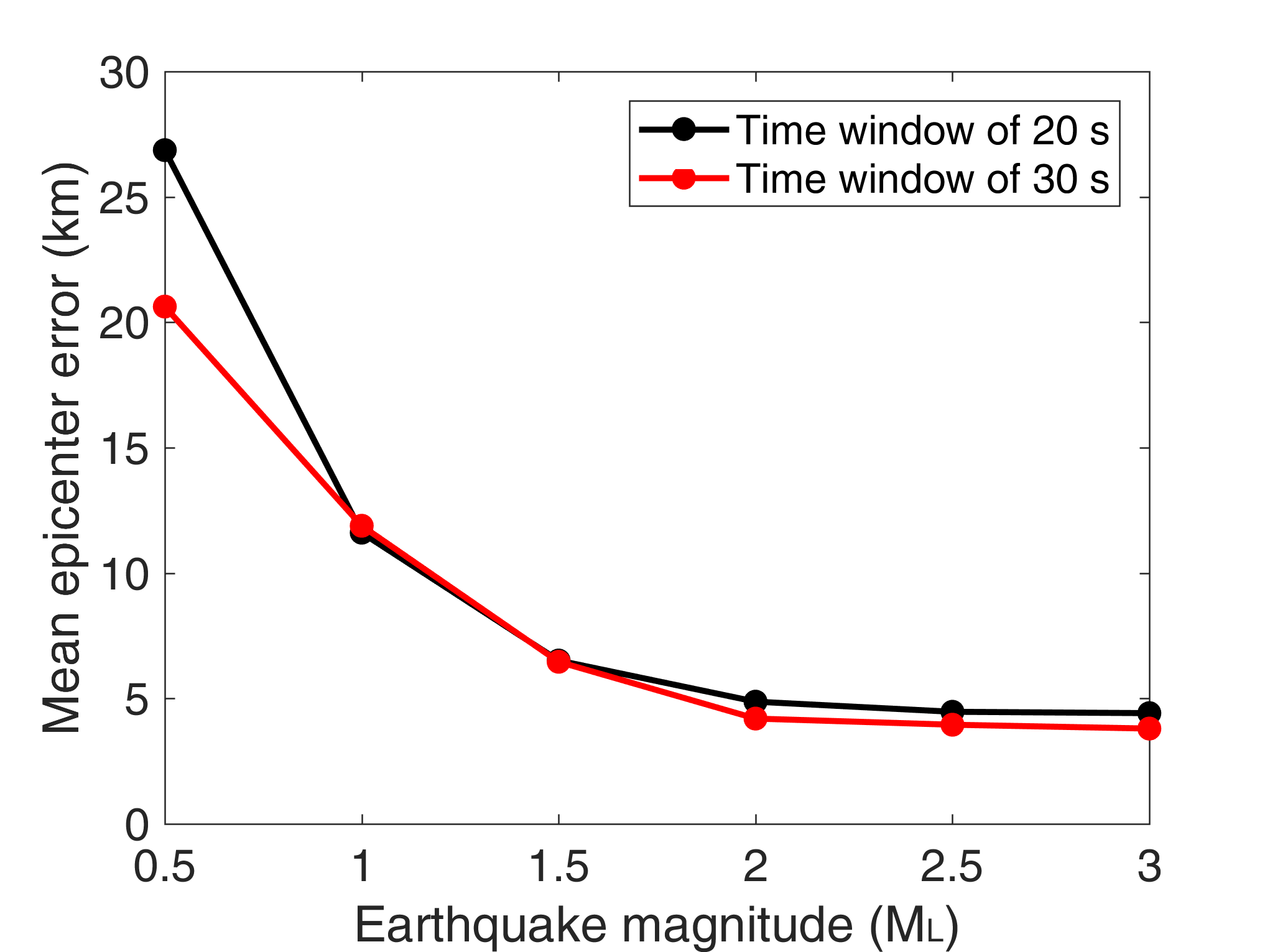


**Fig. S5:** The mean epicenter errors of the prediction with short time windows. The 200 independent testing earthquakes are scaled to different magnitudes, then input the waveforms with different lengths.


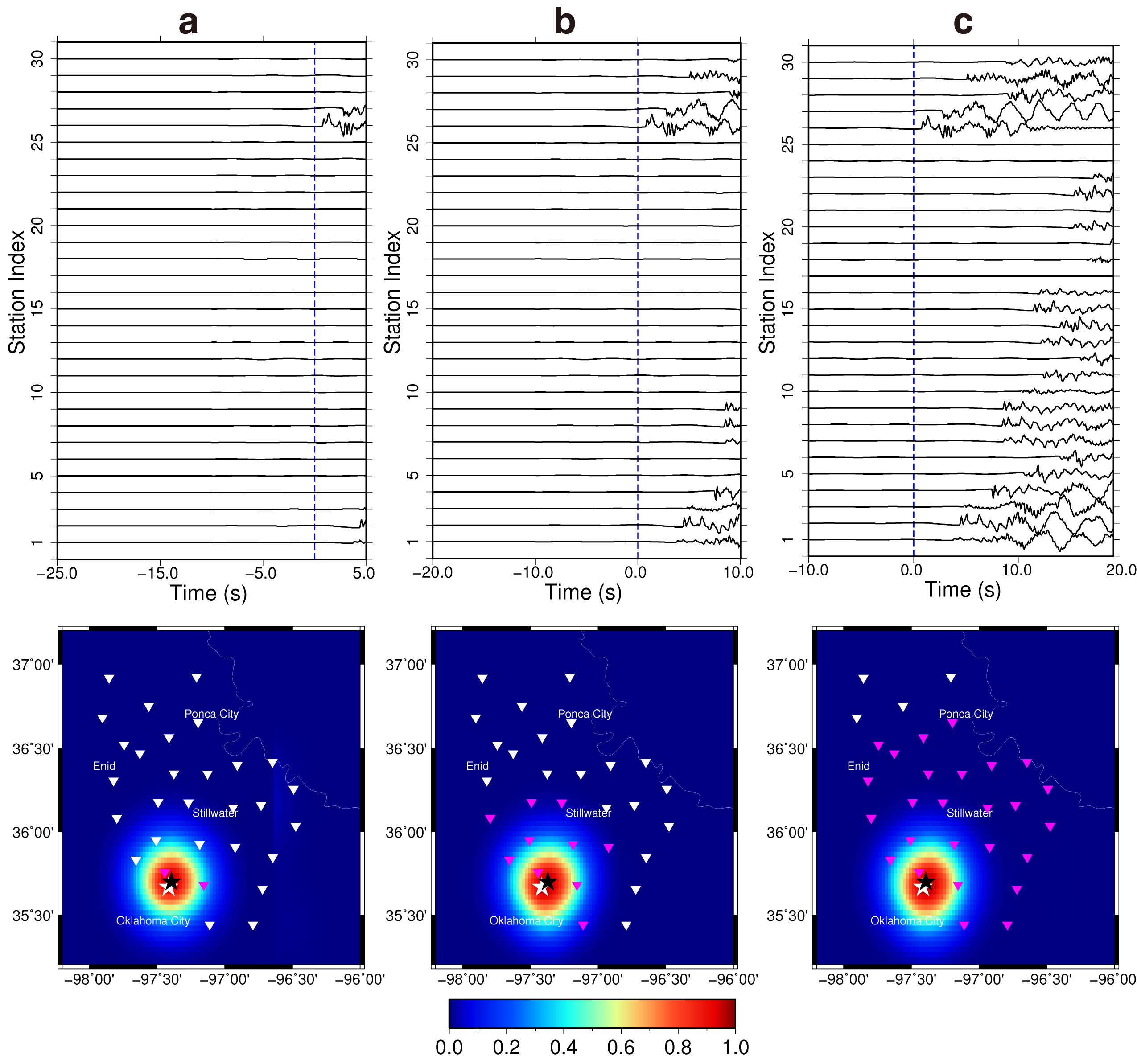


**Fig. S6**: The potential application for earthquake early warning. The location results predicted with an input event of 5 s (A), 10 s (B), and 20 s (C) after the very first arrival. The purple triangles represent the stations receiving data, and the white triangles denote stations without data yet from the event; the black and white stars are the predicted and true locations, respectively; the color image represents the probability values of the event location.


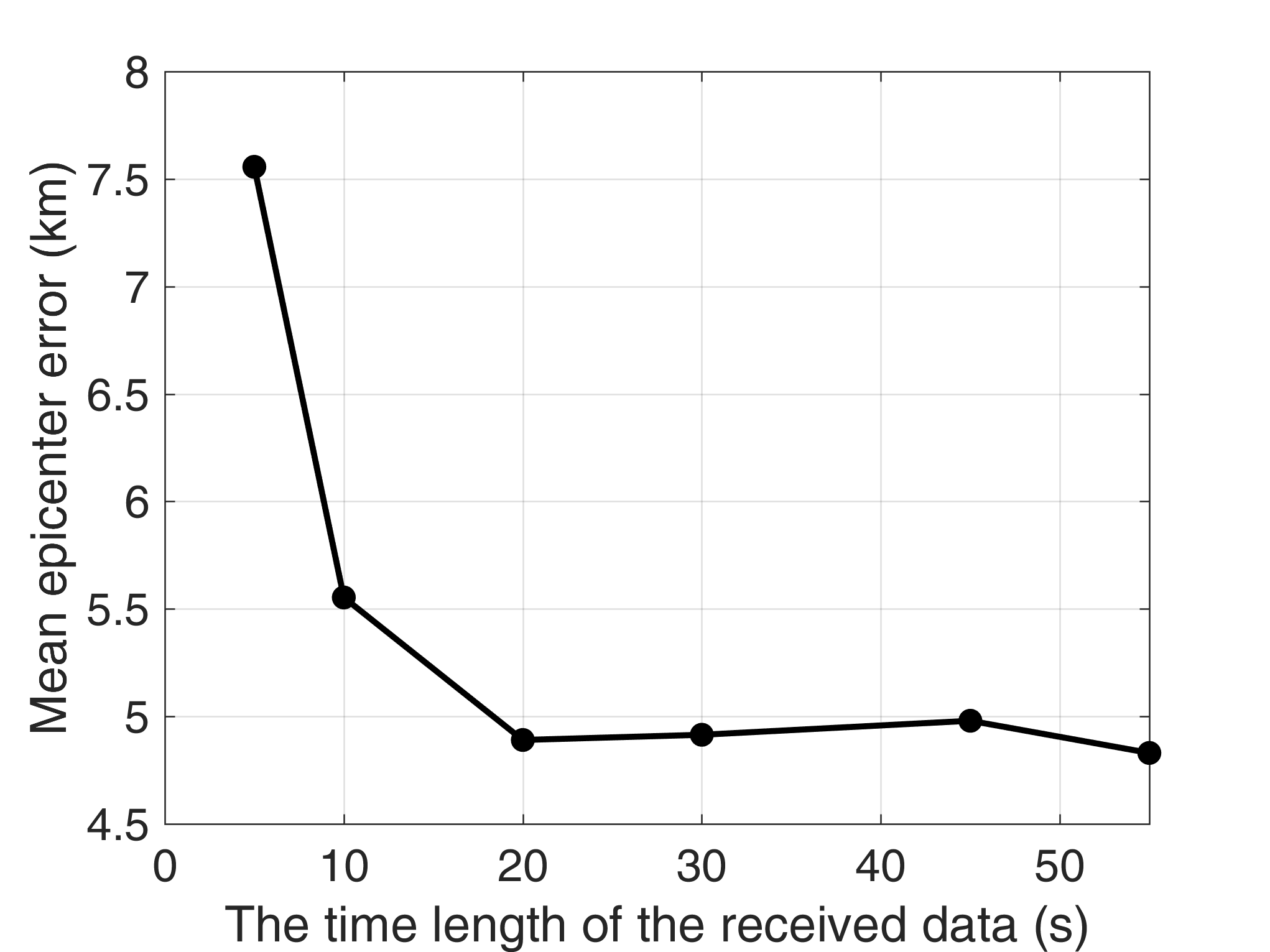


**Fig. S7.** The epicenter error for the prediction with different time length of waveforms. The test set includes 200 earthquakes with different time windows


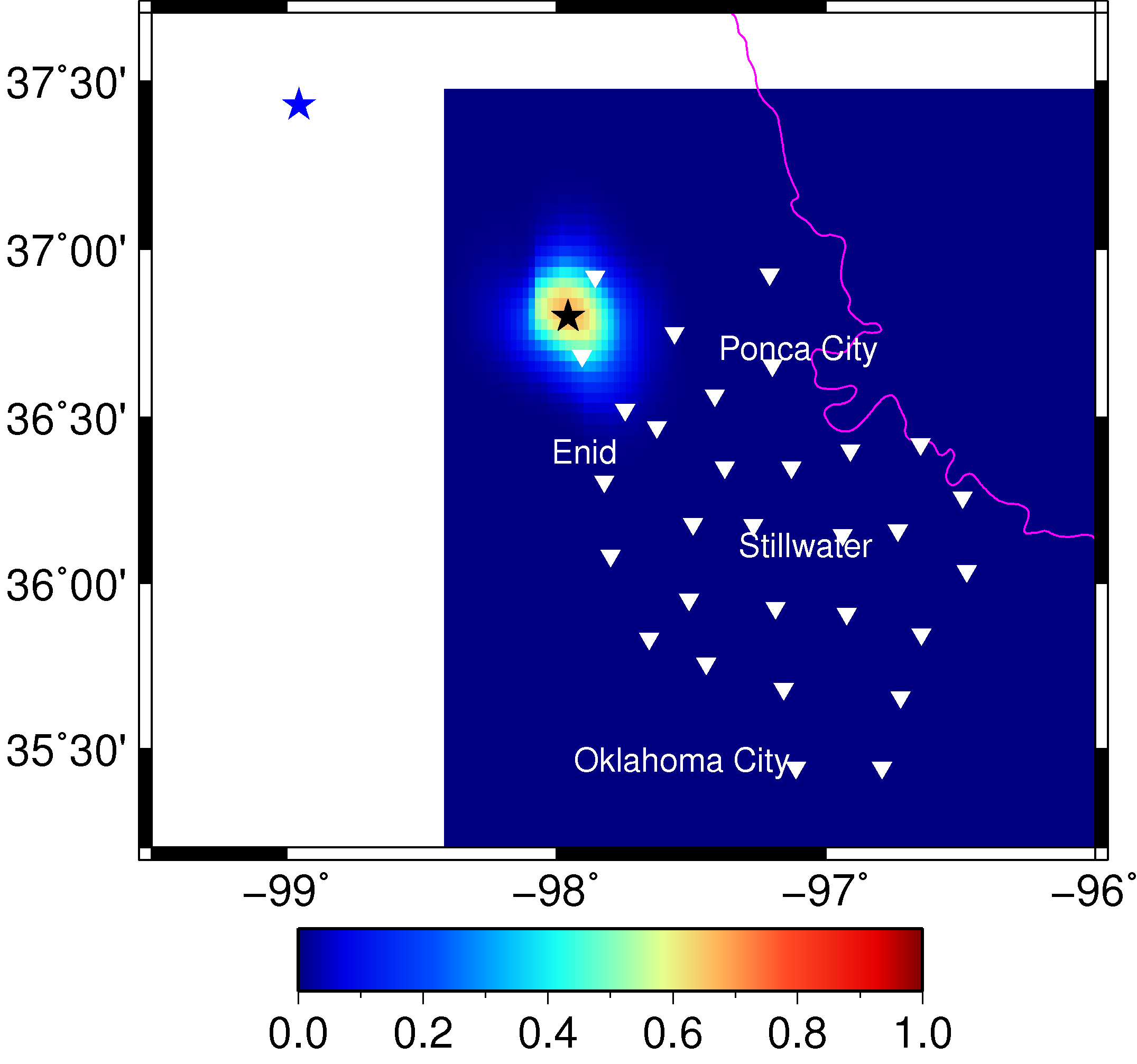


**Fig. S8. The prediction result obtained by inputting the waveform of an earthquake occurring outside the study area.** The maximum value of the Gaussian distribution for the earthquake is approximately 0.6, significantly less than those for the predicted events within the study area.


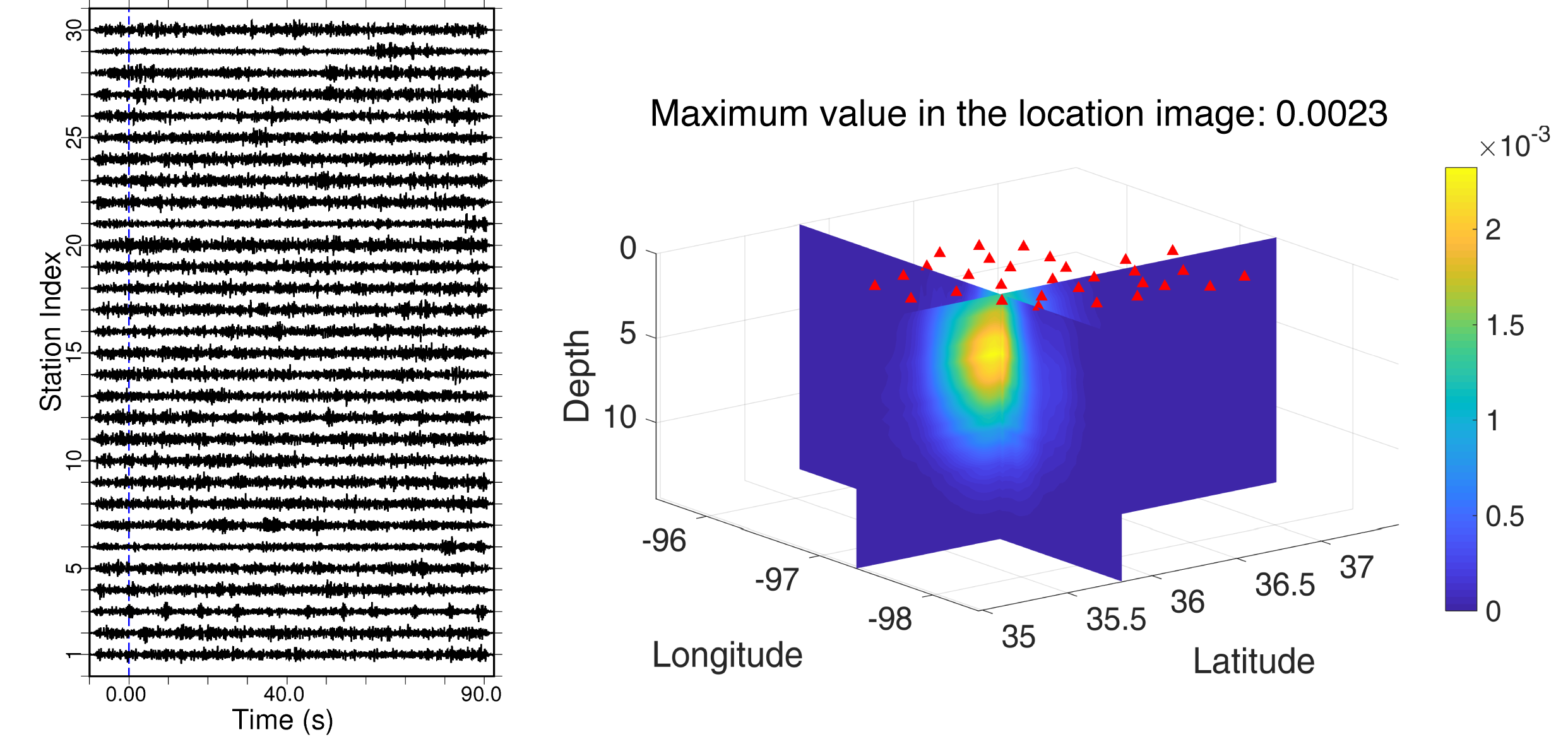


**Fig. S9：**The location result by using noise as input to the neural network. The maximum peak value (0.0023) in the probability image is much less than 1.0 in a normal Gaussian distribution.


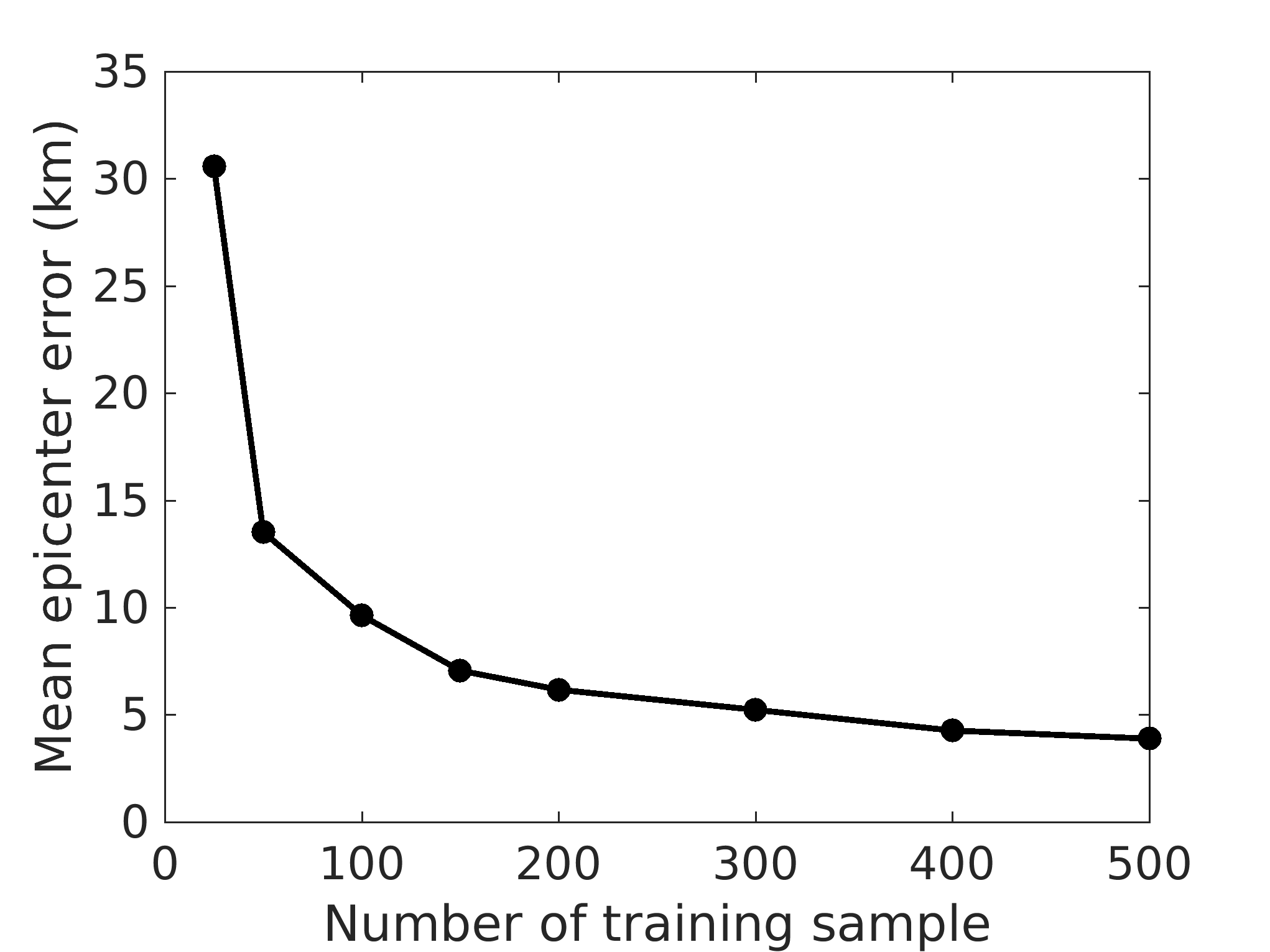


**Fig. S10**: The prediction error by the neural networks trained with different number of training samples and fixed 500 testing samples.


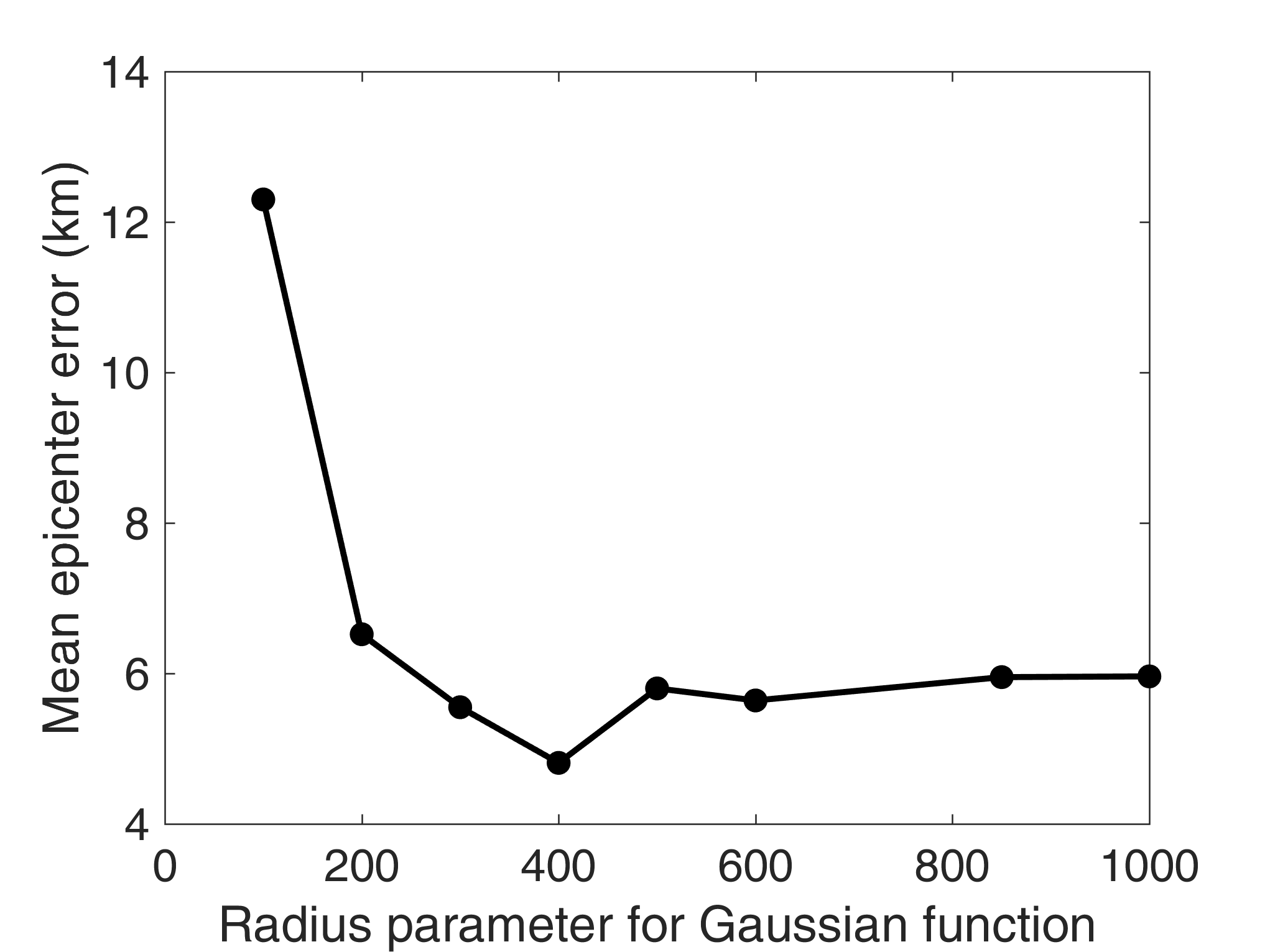


**Fig. S11:** The parameter selection for the Gaussian function when labeling the locations. A large parameter means the radius of the Gaussian function is large. The neural networks are trained with different parameters, and mean epicenter errors are calculated for the 200 independent testing samples. There are 500 samples are used for training for each test.


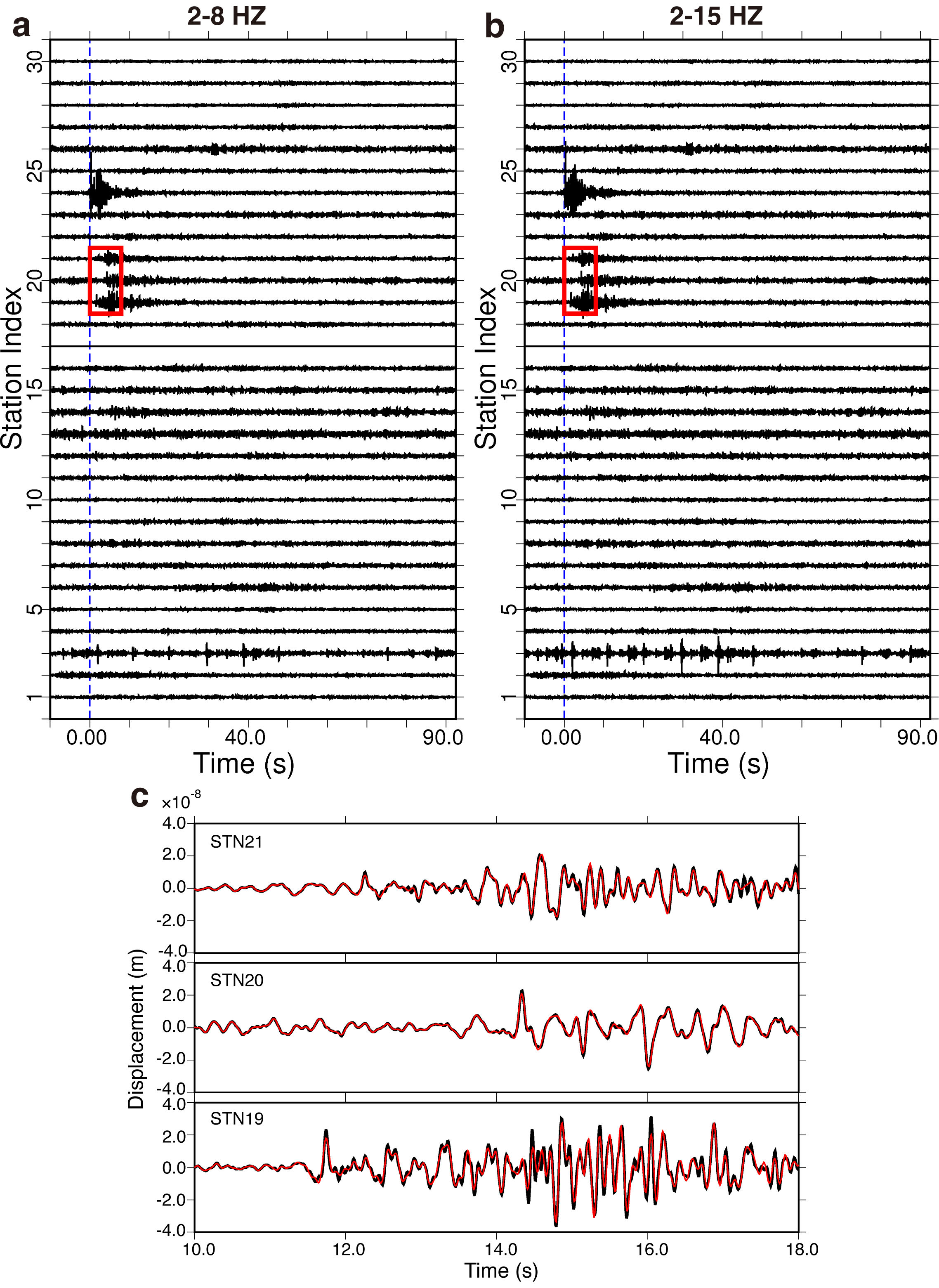


**Fig. S12:** Comparison of the vertical displacement records at three stations due to two different bandpass filters applied to the same event (M_L_ 1.5) on 30 November 2015. (a) Data filtered by a bandpass of 2-8 Hz. (b) Data filtered by a bandpass of 2-15 Hz. (c) The true amplitude display of the same events from the red box in (a) and (b) with 2-8 Hz (red) and 2-15 Hz (black) bandpass filtered applied.

**References**

1. Zhang, H.-M., Chen, X.-F. & Chang, S. An efficient numerical method for computing synthetic seismograms for a layered half-space with sources and receivers at close or same depths. *Pure Appl. Geophys.* **160**, 467-486 (2003).
2. Herrmann, R. B., Benz, H. & Ammon, C. Monitoring the earthquake source process in North America. *Bull. Seismol. Soc. Am.* **101**, 2609-2625 (2011).
3. Vaezi, Y. & Baan, M. V. Comparison of the STA/LTA and power spectral density methods for microseismic event detection. *Geophys. J. Int.* **203**, 1896-1908 (2015).
